# A plasmid toolbox for the easy autodisplay of recombinant proteins and its optimization

**DOI:** 10.1038/s42003-026-10324-7

**Published:** 2026-05-21

**Authors:** Christoph Furtmann, Philip Röhe, Katrin Gesing, Hanna Kuss, Florian Lenz, Joachim Jose

**Affiliations:** University of Münster, Institute for Pharmaceutical & Medicinal Chemistry, Münster, Germany

**Keywords:** Assay systems, Biochemistry, Biological techniques, Molecular biology

## Abstract

Surface display of proteins on bacteria bears several advantages for binding studies, library screening or enzyme inhibitor testing. Here, we present a set of plasmids for autodisplay-based surface expression of recombinant proteins, named **A**utodisplay-**T**ool**B**ox (ATB). In this set, crucial parts as required for autodisplay, including promotor, SP, linker and β-barrel can be combined in varying permutations to find the best combination for a certain protein. The plasmids can be applied individually or in library approaches, enabling optimization of surface display in a single step. By such a library approach, the activity of surface-displayed β-glucosidase (β-Gluc) is increased by a factor of 4.9, the activity of a laccase (CotA) by a factor of 4.7 and the binding capacity of the surface-displayed nucleotide binding domain of human HCN2 channel (HCN2-CNBD) by a factor of 10.3. It is shown that the ATB can be used in different strains of *E. coli* as well as in *Pseudomonas putida*. The aim is to provide a selection of plasmids, accessible by Addgene, that every scientist can use for their own protein, either individually based on personal preferences, structural features, etc., or as a library, as shown here for three different examples.

## Abstract (web summary)

A toolbox of 81 plasmids, accessible by Addgene, facilitates tailor-made autodisplay of proteins in bacteria, e.g. E. coli or P. putida. Plasmids can be applied individually or as a library for optimal promotor, SP, linker, and β-barrel combination.

## Introduction

The development of phage display and its application for the design of peptides and antibodies was awarded the Nobel Prize in chemistry 2018 to George Smith and Gregory Winter^1–3^. Bacterial display can be used similarly for the screening of protein and peptide libraries as well as for the identification of protein ligands. Besides the option to create a new whole-cell biocatalyst by displaying enzymes, bacterial display could have advantages with regard to phage display in the size of the protein displayed, in the number of displayed proteins, and in the applicable screening methods. Moreover, a selected variant displayed on a bacterium can be multiplied without further ado, in contrast to phage display, for which repeated cycles of infection and selection are required to enrich a preferred variant^4^.

For Gram-negative bacteria, like *E. coli*, a simple method for the surface display of recombinant proteins is based on the secretion mechanism of type Va autotransporter (AT) proteins, the so-called classical AT. In analogy, this method was referred to as autodisplay^5,6^.

**Fig. 1:**
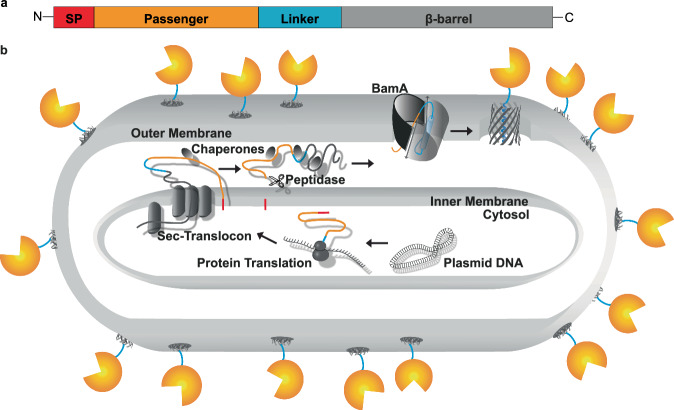
Schematic structure of a classical AT protein & type Va secretion pathway in Gram-negative bacteria. **a** A classical AT of the type Va secretion pathway comprises a N-terminal SP, a passenger domain, a linker and a β-barrel. **b** After the AT is synthesized as precursor in the cytosol, the SP enables Sec translocon mediated transport to the periplasm. During the transport SP is removed. While keeping the AT unfolded, chaperones target the AT to the BAM-Complex^9,10^. BAM assists the folding and outer membrane integration of the AT β-barrel *via* the formation of a hybrid-barrel with BamA. Through the BamA segment of the hybrid-barrel, the passenger domain is translocated to the cell surface in a C- to N-terminal direction. This is initiated by the formation of a C-terminal hairpin. The folding of the C-terminal segments on the cell surface is the key driving force for the translocation of the remaining passenger across the outer membrane^11^. The passenger domain remains attached to the cell surface by the β-barrel, connected *via* the linker. Created with Adobe Illustrator Artwork 16.0 (Dublin, Ireland).

Classical ATs are synthesized as single polydomain/polyprotein precursors that share structural features: a signal peptide (SP) at the N-terminus, followed by a native passenger domain, a linker that usually contains at least one α-helix, and a β-barrel at the C-terminus^4,7^ (Fig. 1a). The SP is cleaved off during the Sec translocon-dependent transport across the inner membrane^8^. Several chaperones (e.g. Skp, SurA) support the transit of the unfolded AT in the periplasm to the β-barrel assembly machinery (BAM)^9,10^. BAM promotes the folding and outer membrane integration of the β-barrel, most probably *via* the formation of a hybrid-barrel with BamA. The passenger domain is translocated through the BamA segment of the hybrid-barrel in a C- to N-terminal direction^11^. This is initiated by the formation of a C-terminal hairpin. The folding of the C-terminal segments on the cell surface is the key driving force for the translocation of the remaining passenger across the outer membrane. The folded passenger remains C-terminally connected to the β-barrel, which functions as a surface anchor^12^ (Fig. 1b). It is worth emphasizing that besides this concept, other models are discussed on β-barrel assembly in the outer membrane^13^. For autodisplay, the protein of interest is replacing the native passenger in the polyprotein precursor. Crucial points for autodisplay are that a host strain for autodisplay needs to be outer membrane protease T (OmpT) negative (e.g. *E. coli* BL21, *E. coli* UT5600), and that the passenger domain is devoid of autoproteolytic capacity to prevent release of the passenger from the surface^4^.

In previous studies, different domains of AT fusion proteins (AT-FP) were separately investigated and optimized to improve surface display of target proteins, e.g. the linker^14–16^ or the β-barrel^17–21^. Additionally, hierarchical strategies were applied by optimizing first the SP and subsequently the β-barrel^22^ or first the SP, subsequently the linker, the β-barrel and finally the promoter^23^. Up to now, combinatorial approaches were exclusively applied to improve features of the recombinant passenger displayed^14,16,18,24–31^. Moreover, bacterial surface display was recently combined with phage display to identify a target protein with improved affinity out of a combinatorial library, supported by deep sequencing^32^. Combinatorial cloning strategies, to permutate AT domains like SP, linker and ß-barrel, however, have not been applied yet.

The present study aimed first to provide a set of plasmids for a customer-made surface display of a protein under investigation. For this purpose, the individual plasmid can be selected based on protein-specific requirements, ordered *via* Addgene and used in a single protein – single plasmid combination. Second, it was the aim to present two library approaches that will enable to find the optimal permutation of the relevant domains for an improved surface display of a certain protein of interest. The two library approaches were not supposed to present a general cloning strategy, but rather a way to find the best combination of SP, linker and β-barrel (and if desired an adequate promotor) for a certain protein to be displayed at the surface of a Gram-negative bacterium, with the significant advantage to provide this in a single step.

Therefore, we present a set of plasmids named the **A**utodisplay-**T**ool**B**ox (ATB), that enables the combination of a classical AT β-barrel with four different SPs and six different linkers under expression control of three different promoters in order to find the optimal set for a given passenger. This set was extended by three variants with an inversed AT β-barrel. An individual plasmid of the ATB (pATB) can be picked for the surface display of distinct passengers based on structural considerations in a rational approach. Moreover, in case the rational approach is not successful, a method to screen the complete set of pATBs as a library is provided to identify the best combination with regard to surface display of functional passengers.

For a first approach, β-glucosidase BglA LYTH from *Caldicellulosiruptor saccharolyticus* (β-Gluc) was taken as a model passenger. It has a size of 54,8 kDa and is in a similar range as many other recombinant passengers expressed by autodisplay before on the cell surface^28,33,34^. In a first embodiment, the pATBs were applied individually, i.e. in a step-by-step approach for obtaining maximum activity of β-Gluc. In a second embodiment, all plasmids were applied as a library, starting either on the DNA level, named all-in-one approach, or starting with strains containing all the different plasmids, named strain-mix approach and screened towards maximal β-Gluc activity. For validation, two further proteins, laccase CotA from *Bacillus coagulans,* as well as the C-linker and cyclic nucleotide binding domain of human hyperpolarization-activated and cyclic nucleotide-gated ion channel (HCN2-CNBD), were subjected to the strain-mix ATB screening. First-time surface display of HCN2-CNBD was achieved, as well as a substantial improvement of the activities of both proteins on the cell surface.

## Results

### AT-FP with classical composition for the ATB

**Fig. 2:**
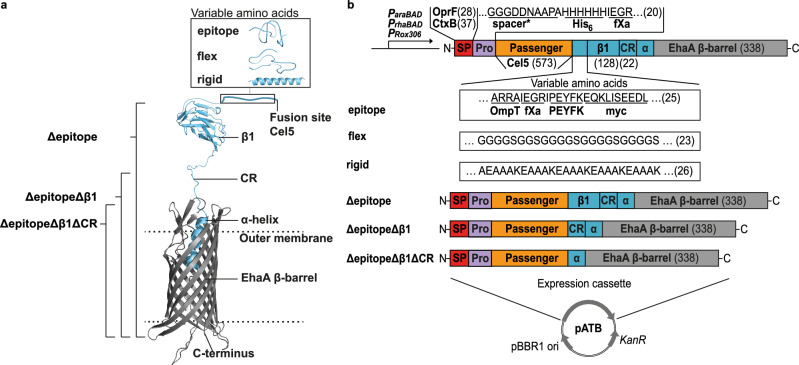
Set of ATB AT-FP variants with a classical AT β-barrel. **a** The secondary structures of the EhaA β-barrel, the CR, and the β1 domain were modeled with RaptorX^80^ and the variable amino acids in the linker with PEP-FOLD3^79^. Deletion variants of the EhaA linker are denoted. **b** Classical AT-FP contained N-terminal either a CtxB or an OprF SP. The pro-region started after the SP and optionally (*) contained a sequence of EstA functioning as a spacer in addition to an obligatory His_6_-tag and a fXa protease cleavage site. Linker (light blue) variants with full length contained a sequence of variable amino acids, a β1 domain, a CR and the α-helix of the EhaA β-barrel. The variable amino acids were either a combination of protease cleavage sites (OmpT, fXa) and tags (PEYFK, myc) or the flexible, disordered structure forming G_4_S-G_2_S-(G_4_S)_3_ sequence or the rigid, α-helical structure forming A(EAAAK)_5_ sequence (called: rigid). Linker deletion variants contained either a β1 and a CR or only a CR or Cel5, which was directly fused to the α-helix of the EhaA β-barrel. The number of amino acids of the corresponding part of the AT-FP is given in brackets. The plasmids were constructed based on the MATE plasmid backbone^44^. AT-FP expression was set under control of *P*_*araBAD*_, *P*_*rhaBAD*_ and *P*_*Rox306*_. Created with Adobe Illustrator Artwork 16.0 (Dublin, Ireland).

The AT-FPs applied for the ATB (Fig. 2a, b) contained either an SP from the cholera toxin B subunit (CtxB) from *Vibrio cholerae*^35^ or an SP from the outer membrane porin F (OprF) from *Pseudomonas putida* KT2440^36^. The so-called pro-region – 30 aa downstream the SP cleavage site – was previously shown to affect secretion efficiency^37,38^. The first ten aa (GGGDDNAAPA) of the pro-region of Esterase A from *Burkholderia gladioli* (EstA)^39^ appeared to be beneficial for surface display *via* AT-FP with both CtxB and OprF SP^22^. Hence, these ten aa were taken as an insertion option instead of the complete EstA pro-region and termed ‘spacer’. The pro-region was complemented by an obligatory 6x histidine-tag (His_6_-tag) and an obligatory factor Xa (fXa) protease cleavage site. Behind this artificial pro-region, initially, the passenger domain Cel5 from *Hahella chejuensis* (Uniprot ID: Q2SFD8) was inserted, followed by one of six different linkers between the passenger and the AT β-barrel. The first linker variant (called: epitope) contained an OmpT & a factor Xa protease cleavage site, a linear tag derived from HIV Nef protein (PEYFK)^5^ and a myc-tag. The epitope linker variant was complemented by two native domains of the EhaA AT, the β1 domain and a connecting region (CR)^40^. For the epitope linker, a random coil structure was predicted. In a second variant (called: flex), instead of the epitope linker, the aa sequence G_4_S-G_2_S-(G_4_S)_3_ was inserted, which forms a random coil structure, going along with high flexibility^41^. In a third variant (called: rigid), the aa sequence A(EAAAK)_5_ replaced the epitope linker, which was described to form an α-helical structure^42^, going along with more rigidity^43^. Three deletion linker variants contained either only the β1 and the CR part (called: Δepitope) or the CR part alone (called: ΔepitopeΔβ1) or finally no linker at all, i.e. Cel5 was directly attached to the β-barrel (called: ΔepitopeΔβ1ΔCR). All variants as described were used with the EhaA AT β-barrel, which comprised an additional α-helix^40^ required for plugging the β-barrel after transport.

Plasmids encoding the AT-FP variants (Table 1) were individually constructed based on the plasmid backbone for **m**aximized **a**uto**t**ransporter-mediated **e**xpression (MATE)^44^, which is established for autodisplay^45^. To optimally control the expression level of recombinant proteins, a tunable promoter is of importance^4,46–48^. Therefore, AT-FP expression was set under control of an arabinose inducible promoter *P*_*araBAD*_, a rhamnose inducible promoter *P*_*rhaBAD*_, and the cell density-dependent, auto-inducible promoter *P*_*Rox306*_, which were all proven suitable for autodisplay before^22,45,46,49^.

**Table 1:** Plasmids of the ATB





*The DNA backbone sizes refer to the respective plasmid without initial passenger DNA cel5G which itself was not part of the classification.

**All YeeJ AT-FP variants contained the SP native to YeeJ.

This library of 72 plasmids was termed ATB, with each pATB obtaining a specific three-digit identification code (xyz), denoting the type of promoter (1yz – 3yz, Table 1, green), SP +/- spacer (x1z – x4z, Table 1, red), the type of linker (xy1 – xy6, Table 1, blue).

### Step-by-step preparation of a pATB*-β-gluc* library and consecutive screening

**Fig. 3:**
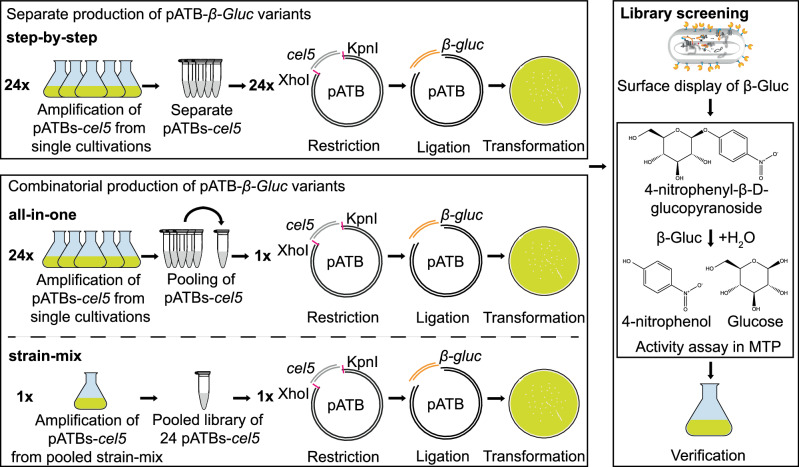
Concepts of different ATB-based library screenings. **Step-by-step**: Individual digest of the 24 pATB-*cel5* with XhoI/KpnI removed the initial passenger DNA *cel5*. After separate ligation of pATB DNA backbone and the *β-gluc* DNA insert for each variant, the 24 pATB*-β-gluc* were separately produced in *E. coli* DH5α, and the corresponding collection of plasmids was termed pATB*-β-gluc* step-by-step library. **All-in-one**: the 24 pATB-*cel5* plasmids were manually pooled, digested with XhoI/KpnI to remove the initial passenger DNA sequence *cel5*. Ligation of the pATB DNA backbone and the *β-gluc* DNA insert resulted in the combinatorial pATB*-β-gluc* all-in-one library. **Strain-mix**: the 24 pATB-*cel5* plasmids were isolated from a mixture of strains that were previously transformed with 24 pATB-*cel5* plasmids. Subsequently, the plasmid preparation containing the 24 variants was digested with XhoI/KpnI to remove the initial passenger DNA sequence *cel5*. Ligation of the resulting pATB DNA backbones and the *β-gluc* DNA insert resulted in the combinatorial pATB*-β-gluc* strain-mix library. The all-in-one and the strain-mix libraries were used to transform *E. coli*. **Library screening**: *E. coli* pATB*-β-gluc* variants were cultivated and screened in a 96-well MTP with *p*NPG as substrate. The highest performing variants were verified in a 0.2 L scale. Created with Adobe Illustrator Artwork 16.0 (Dublin, Ireland).

**Fig. 4:**
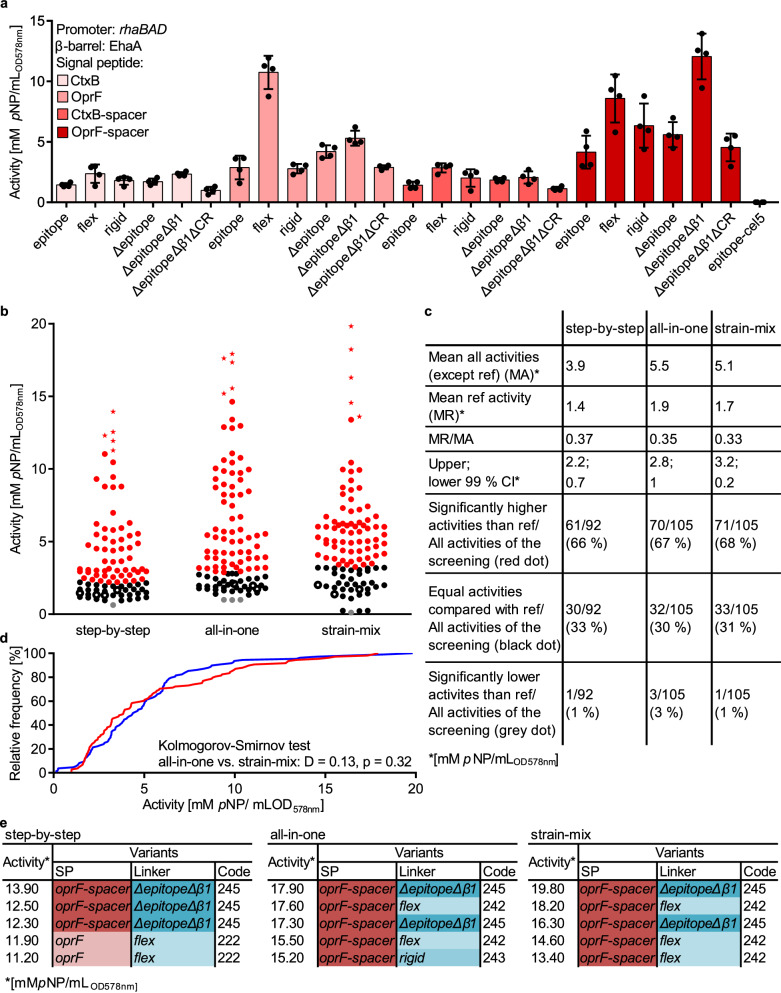
β-Gluc activities as obtained with different pATB libraries. **a** The mean activity of β-Gluc displaying cells in the step-by-step library screening assigned to the respective variants. Each variant contained the EhaA β-barrel and the expression was under the control of *P*_*rhaBAD*_. The SP is color-coded and the x-axis label denotes the type of linker. Cells displaying cel5 (epitope-cel5) encompassed a CtxB SP and served as control. The cells were incubated with 5 mM *p*NPG, and turnover was detected by measurement of *p*NP absorption at 405 nm after 9 min reaction time (*n* = 4 biologically independent samples) (**b**) each dot represents the activity either of the reference *P*_*rhaBAD*_*-ctxB-β-gluc-epitope-ehaA* or of variants from one of 92 (step-by-step) or 105 (all-in-one & strain-mix) 96-well MTP scale cultivations with subsequent *p*NPG assay as described in a). The 99% CI of the reference mean activity (MR) was used to identify significantly higher (red dots), equal (black dots) and lower (gray dots) activities than the reference (black circles). The five highest activities of each screening are marked (stars). The quotient of MR and the mean of all activities except the reference (MA) served to compare the different screening approaches. **a** Comparison of screening results of the pATB libraries by quantification of different parameters. ref = reference. **d** A K-S test was performed with the cumulative frequency distributions of the all-in-one (red) and strain-mix (blue) screening data sets. **e** The plasmids harbored by the five variants with the highest activity of each library screening were assigned to the respective code (step-by-step) or subjected to sequence analysis (all-in-one & strain-mix) to identify the corresponding SP and linker.

For the proof of concept, pATBs were tested with β-Gluc^27^ as a model passenger in *E. coli*. For this purpose, in each single plasmid, the initial *cel5* passenger was replaced by *β-gluc* using restriction/ligation by the KpnI/XhoI sites, resulting in 24 pATB-*β-gluc* variants, called step-by-step variants (Fig. 3). Each pATB was used to transform *E. coli*, resulting in 24 strains derived from single colonies. Four randomly chosen single colonies per different variant and a reference were tested for activity. As a reference, *P*_*rhaBAD*_*-ctxB-β-gluc-epitope-ehaA* (pATB_211-*β-gluc*) was selected, as it contained the combination of CtxB SP and epitope linker frequently used for bacterial surface display^15,22,44,45,50–52^. Cells were cultivated in a 200 µL scale in 96-well microtiter plates (MTPs) overnight and subjected to an activity assay with 4-nitrophenyl-β-D-glucopyranoside (*p*NPG) as substrate and 4-nitrophenol (*p*NP) as product (Fig. 4a). Control cells expressing *P*_*rhaBAD*_*-ctxB-cel5-epitope-ehaA* (pATB_211-*cel5*) showed neglectable activity (0.00625 mM *p*NP/mL_OD578nm_), whereas each variant, supposed to have β-Gluc as passenger, showed an activity > 0.00625 mM *p*NP/mL_OD578nm_. This result indicated all plasmids were functional in expressing a corresponding AT-FP gene, but with different enzymatic activities. Compared to the ref (1.47 mM *p*NP/mL_OD578nm_) the highest activity was shown by the variants *P*_*rhaBAD*_*-oprf-spacer-β-gluc-rigid-ehaA* (pATB_243-*β-gluc*) with 6.34 mM *p*NP/mL_OD578nm_, *P*_*rhaBAD*_*-oprF-spacer-β-gluc-flex-ehaA* (pATB_242-*β-gluc*) with 8.59 mM *p*NP/mL_OD578nm_, *P*_*rhaBAD*_*-oprF-β-gluc-flex-ehaA* (pATB_222-*β-gluc*) with 10.75 mM *p*NP/mL_OD578nm_ and *P*_*rhaBAD*_*-oprF-spacer-β-gluc-ΔepitopeΔβ1-ehaA* (pATB_245-*β-gluc*) with 12.06 mM *p*NP/mL_OD578nm_. In summary, the pATBs appeared to be suitable to screen for an optimal combination of SP and linker for a distinct passenger and host. Nevertheless, the step-by-step approach to constructing variants before screening appeared laborious and time-consuming. In consequence, two easier approaches were investigated.

### All-in-one & strain-mix preparation of pATB*-β-gluc* libraries for screening

In the combinatorial all-in-one cloning approach to prepare a pATB library for screening for activity, the 24 ATB plasmids with *cel5* as passenger (pATB-*cel5*) were manually pooled in equimolar concentrations (Fig. 3). The pooled plasmids were subjected to a restriction digest with XhoI/KpnI and the DNA fragments were treated with alkaline phosphatase (FastAP) to prevent religation. Likewise, XhoI/KpnI was used to treat a plasmid carrying the *β-gluc* DNA insert sequence (p*β-gluc*). For ligation, the molar ratio of insert to backbone was set to 3:1, corresponding to approx. 12 fmol DNA backbone in total ( = 60 ng DNA considering an average backbone size of 8152 bp) and approx. 36 fmol ( = 30 ng DNA considering an insert size of 1366 bp) of the *β-gluc* DNA insert. The resulting pATB*-β-gluc* all-in-one library was used to transform *E. coli* (single-cell clone library size: 305). Finally, 105 clones were tested for activity, representing a 98.5% statistical coverage of all possible 24 variants (calculated with GLUE-IT CASTER 2.0^53^).

All picked clones showed an activity >0 mM *p*NP/mL_OD578nm_ (Fig. 4b), indicating that the digest of the pooled pATBs was successful and no ancestor pATB-*cel5* was included in the screening. Nevertheless, the all-in-one approach could have potentially biased the screening result compared to the step-by-step approach. This bias could have been due to distinct pATB-*cel5* variants that have been preferably addressed in the pooled restriction digest, favored ligation of particular pATB DNA backbones in the following ligation step, different transformation efficiencies of the pATB*-β-gluc* or hampered growth of the freshly transformed *E. coli* cells. To quantify a potential bias, the result of the all-in-one screening was compared with the result of the step-by-step screening juxtaposed as a dot plot (Fig. 4b, c). The quotient *mean of reference activity/mean of all activities* (*MR/MA*) was similar for the step-by-step (0.37) and the all-in-one (0.35) after screening. Therefore, the difference in the MA was attributed to deviations between individual screening runs and was not considered any further. The 99% confidence interval (CI) of MR served as a threshold to identify single-cell clones with significant differences compared to the ref *P*_*rhaBAD*_*-ctxB-β-gluc-epitope-ehaA* (Fig. 4b, c). 66% (step-by-step) or 67% (all-in-one) of the single-cell activities were considered as significantly higher (red dots), 33% (step-by-step) or 30% (all-in-one) of the activities were considered as equal (black dots), and 1% (step-by-step) or 3% (all-in-one) of the activities were considered as significantly lower (gray dots) than the activity of the ref (black circles). Sequence analysis of the plasmids from the five variants with the highest activity (Fig. 4b, stars) in the all-in-one screening unveiled variant *P*_*rhaBAD*_*-oprf-spacer-β-gluc-rigid-ehaA* (243), *P*_*rhaBAD*_*-oprF-spacer-β-gluc-flex-ehaA* twice and *P*_*rhaBAD*_*-oprF-spacer-β-gluc-ΔepitopeΔβ1-ehaA* (245) twice (Fig. 4e). Two of them, *P*_*rhaBAD*_*-oprf-spacer-β-gluc-rigid-ehaA* (243) and *P*_*rhaBAD*_*-oprF-spacer-β-gluc-ΔepitopeΔβ1-ehaA* (245), have been identified by the step-by-step approach as well. This means that both library approaches applied to consecutive screening yielded a similar distribution pattern of activity values, a similar rate of significantly different activities compared to the reference and led to an identical best-performing variant in the activity assay (variant *P*_*rhaBAD*_*-oprF-spacer-β-gluc-ΔepitopeΔβ1-ehaA* (245)) (Fig. 4b, c, e). In consequence, based on the compared parameters (Fig. 4c), the simplified library preparation *via* the all-in-one approach appeared not to bias the screening result.

To go a step further, a bacterial strain-mix approach was investigated to accomplish a fast, on-demand library amplification. The idea was to amplify the pATB-*cel5* library from transformed, cryo-preserved stocks of *E. coli* DH5α. For this purpose, a mixture of 24 manually pooled pATB-*cel5* plasmids was used as a whole to transform competent *E. coli* DH5α cells (single-cell clone library size: 298). All single-cell clones were taken up from the agar plates with 5 mL lysogeny broth (LB) medium containing 50 μg mL^−1^ kanamycin (Kan50), mixed with glycerol (25% v/v final concentration) and stored as a cryo-preserved cell stock in aliquots of 0.5 mL. An aliquot of this stock was used to inoculate an overnight culture. Subsequently, the plasmids were isolated from this overnight culture, subjected to restriction digestion (XhoI/KpnI) and ligated with a similarly digested *β-gluc* fragment. Finally, the resulting new pATB-*cel5* strain-mix library was screened according to the protocol as described for the all-in-one approach. When the screening results of this strain-mix approach were juxtaposed with the results of the step-by-step approach (Fig. 4b, c, e), it turned out, that their *MR/MA* values were similar (strain-mix: 0.33, step-by-step: 0.37), they had a similar distribution pattern of activities, a similar amount of activities that were significantly higher compared to the reference (strain-mix: 68%, step-by-step: 66%) and again led to the identical best-performing variant as before, namely variant *P*_*rhaBAD*_*-oprF-spacer-β-gluc-ΔepitopeΔβ1-ehaA* (245).

As a further approach to quantify or exclude a potential bias, a Kolmogorov-Smirnov (K-S) test was performed with the cumulative frequency distributions of the all-in-one (Fig. 4d, red) and strain-mix (Fig. 4d, blue) screening data sets. This resulted in a K-S D-value of 0.13 and a p-value of 0.32, indicating that all-in-one and strain-mix were sampled from identical distributions and hence, no bias appeared to be present. When a similar approach was applied to the cumulative frequency distributions of the step-by-step screening data set in comparison to the all-in-one (K-S *D* = 0.25, *p* = 0.004) and the strain-mix (K-S *D* = 0.35, *p* < 0.0001) data sets, it turned out that most probably, they were sampled from not identical distributions, and in consequence, a bias could not be excluded. However, it was beyond our available means to quantify this bias any further. For future approaches in applying the toolbox, it would be a recommendable option for researchers to use deep sequencing methods, such as Oxford Nanopore Sequencing or Illumina Sequencing, before analyzing a library in terms of enzyme activity or ligand binding. This would allow us to determine a possible bias more precisely, e.g. in terms of library width with interquartile ranges or Shannon-entropy. However, as all parameters as given in Fig. 4c appeared to be similar across the three approaches, and the best-performing variants as identified were identical, such a bias appeared not to be decisive for the overall goal of the pATB screening.

To conclude, the bacterial strain-mix appeared to be suitable for the production of a pATB-*cel5* library that subsequently can be used to prepare a pATB library with a passenger of interest like β-Gluc.

### Analyzing *E. coli* pATB*-β-gluc* variants with highest enzymatic activity

**Fig. 5:**
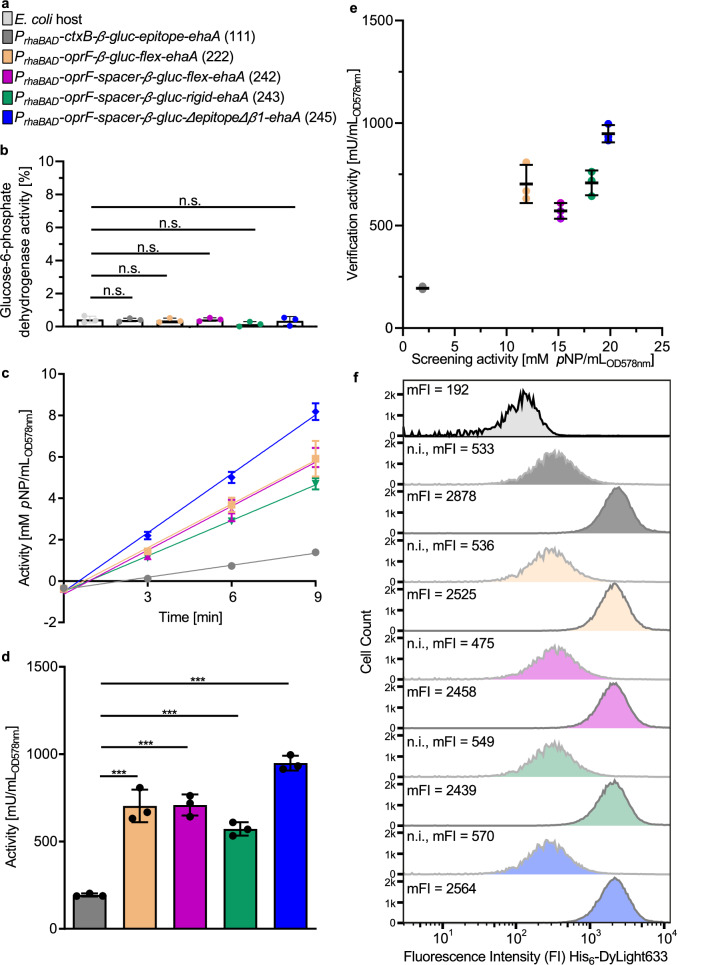
Assessment of cell lysis, β-Gluc activity and level of surface display for best-performing variants after large-scale cultivation. **a** Color coding for the variants analyzed further: *E. coli* pATB*-β-gluc* variants *P*_*rhaBAD*_*-oprF-β-gluc-flex-ehaA* (222), *P*_*rhaBAD*_*-oprF-spacer-β-gluc-flex-ehaA* (242), *P*_*rhaBAD*_*-oprf-spacer-β-gluc-rigid-ehaA* (243), *P*_*rhaBAD*_*-oprF-spacer-β-gluc-ΔepitopeΔβ1-ehaA* (245), the reference *P*_*rhaBAD*_*-ctxB-β-gluc-epitope-ehaA* and *E. coli* host cells. The corresponding pATB code from Table 1 is given in brackets. The color coding is kept constant throughout the entire figure. **b** The lysis of these cells was assessed by measurement of Glc-6-P dehydrogenase activity in the supernatant. To determine 100% Glc-6-P dehydrogenase activity, the supernatant of completely lysed host cells was measured accordingly. The ratio between the Glc-6-P dehydrogenase activity of test samples and of lysed cells was used to calculate the corresponding degree of lysed cells in percent (*n* = 3 biologically independent samples, ±SD, one-way ANOVA, post-test Dunnett’s multiple comparison: n.s. = not significant). **c** Cells were adjusted to a final OD_578nm_ of 0.1, and the activity was determined with 5 mM *p*NPG after 0, 3, 6, and 9 min reaction time by measurement of *p*NP absorption at 405 nm. Linear regression was performed on the produced mM *p*NP/mL_OD578nm_ for the reference (*R*^2^ = 0.98), *P*_*rhaBAD*_*-oprF-β-gluc-flex-ehaA* (222) (*R*^2^ = 0.97), *P*_*rhaBAD*_*-oprF-spacer-β-gluc-flex-ehaA* (242) (*R*^2^ = 0.98), *P*_*rhaBAD*_*-oprf-spacer-β-gluc-rigid-ehaA* (243) (*R*^2^ = 0.99), *P*_*rhaBAD*_*-oprF-spacer-β-gluc-ΔepitopeΔβ1-ehaA* (245) (*R*^2^ = 0.99) (*n* = 3 biologically independent samples, ±SD). **d** Based on the slope of the linear regression from (**c**), the activity of the variants was calculated in mU/mLOD_578nm_ (*n* = 3 biologically independent samples, ±SD, one-way ANOVA, post-test Dunnett’s multiple comparison: ***p ≤ 0.001). **e** The activities of the reference and the variants *P*_*rhaBAD*_*-oprF-β-gluc-flex-ehaA* (222), *P*_*rhaBAD*_*-oprF-spacer-β-gluc-flex-ehaA* (242)*, P*_*rhaBAD*_*-oprf-spacer-β-gluc-rigid-ehaA* (243) and *P*_*rhaBAD*_*-oprF-spacer-β-gluc-ΔepitopeΔβ1-ehaA* (245) obtained in the verification (**d**) were set in relation to the activities of these variants obtained in the screenings (Fig. 4e) (**f**) surface accessibility of β-Gluc was determined by flow cytometry analysis. Protein expression was either induced using 2 mM of L-rhamnose or was not induced (n.i.). The cells were treated with a primary anti-His_6_ antibody and a secondary DyLight-633 conjugated antibody and analyzed via flow cytometry (ex. 633 nm, em. 660 nm) without setting any gate. The FI values of 50,000 cells per variant is shown.

To verify the results of the pATB library screenings in a larger scale, the four variants *P*_*rhaBAD*_*-oprF-β-gluc-flex-ehaA* (222), *P*_*rhaBAD*_*-oprF-spacer-β-gluc-flex-ehaA* (242), *P*_*rhaBAD*_*-oprf-spacer-β-gluc-rigid-ehaA* (243), *P*_*rhaBAD*_*-oprF-spacer-β-gluc-ΔepitopeΔβ1-ehaA* (245), as well as the reference and *E. coli* host without plasmid (blank) were cultivated in 200 mL LB (Fig. 5a). After harvesting, OD_578nm_ was adjusted to 0.1 and a whole-cell activity assay with *p*NPG was performed. Linear regression was performed to the produced mM *p*NP/mL_OD578nm_ for the reference, *P*_*rhaBAD*_*-oprF-β-gluc-flex-ehaA* (222), *P*_*rhaBAD*_*-oprF-spacer-β-gluc-flex-ehaA* (242), *P*_*rhaBAD*_*-oprf-spacer-β-gluc-rigid-ehaA* (243), *P*_*rhaBAD*_*-oprF-spacer-β-gluc-ΔepitopeΔβ1-ehaA* (245) (Fig. 5c). The slope, was used to calculate the activity of the variants in mU/mL_OD578nm_ (Fig. 5d). Compared to the reference (193 mU/mL_OD578nm_), variant *P*_*rhaBAD*_*-oprf-spacer-β-gluc-rigid-ehaA* (243) (571 mU/mL_OD578nm_) showed a 3-fold increased enzyme activity, whereas variant *P*_*rhaBAD*_*-oprF-β-gluc-flex-ehaA* (222) (703 mU/mL_OD578nm_) and *P*_*rhaBAD*_*-oprF-spacer-β-gluc-flex-ehaA* (242) (708 mU/mL_OD578nm_) showed 3.6-fold increased enzyme activities. The highest enzymatic activity of all variants, also after large-scale cultivation, showed variant *P*_*rhaBAD*_*-oprF-spacer-β-gluc-ΔepitopeΔβ1-ehaA* (245) (948 mU/mL_OD578nm_), indicating a 4.9-fold increase compared to the reference.

It was assessed whether variants that appeared to have higher activity in the initial screening were indeed better based in the following orthogonal verification experiment. For this purpose, the activity data obtained in the verification experiment (Fig. 5d) were set into relation to the activity data in the initial screening (Fig. 4e). As can be seen in resulting Fig. 5e, the four variants *P*_*rhaBAD*_*-oprF-β-gluc-flex-ehaA* (222), *P*_*rhaBAD*_*-oprF-spacer-β-gluc-flex-ehaA* (242), *P*_*rhaBAD*_*-oprf-spacer-β-gluc-rigid-ehaA* (243) and *P*_*rhaBAD*_*-oprF-spacer-β-gluc-ΔepitopeΔβ1-ehaA* (245) had clearly higher activity compared to the reference and variant *P*_*rhaBAD*_*-oprF-spacer-β-gluc-ΔepitopeΔβ1-ehaA* (245) was the most active in both, the initial screening as well as in the subsequent orthogonal verification. The second-highest activity in the screening as well as in the verification, showed variant *P*_*rhaBAD*_*-oprF-spacer-β-gluc-flex-ehaA* (242), while variant *P*_*rhaBAD*_*-oprf-spacer-β-gluc-rigid-ehaA* (243) swapped places with variant *P*_*rhaBAD*_*-oprF-β-gluc-flex-ehaA* (222) in the verification.

After SDS-PAGE of outer membrane proteins and subsequent densitometric analysis, the level of surface-displayed β-Gluc was estimated 0.9-fold lower for variant *P*_*rhaBAD*_*-oprF-β-gluc-flex-ehaA* (222), approx. 1.3-fold higher for variants *P*_*rhaBAD*_*-oprF-spacer-β-gluc-flex-ehaA* (242) and *P*_*rhaBAD*_*-oprf-spacer-β-gluc-rigid-ehaA* (243) and approx. 1.9-fold higher for variant *P*_*rhaBAD*_*-oprF-spacer-β-gluc-ΔepitopeΔβ1-ehaA* (245) compared to the reference (Supplementary Fig. 1, Supplementary Fig. 2 and Supplementary Table 1). For a further proof of surface display, cells were treated with a primary anti-His_6_ antibody and a secondary DyLight-633 conjugated antibody and subjected to flow cytometry (Fig. 5f). The mean fluorescence intensity (mFI) of the variants after induction of protein expression was set into relation to mFI of the corresponding cells not induced obtaining the relative mFI (Supplementary Table 2). Based on these data, the level of surface display for the reference was 1.15-fold higher compared to variant *P*_*rhaBAD*_*-oprF-β-gluc-flex-ehaA* (222), 1.04-fold higher compared to variant *P*_*rhaBAD*_*-oprF-spacer-β-gluc-flex-ehaA* (242), 1.22-fold higher compared to variant *P*_*rhaBAD*_*-oprf-spacer-β-gluc-rigid-ehaA* (243) and 1.21-fold higher compared to variant *P*_*rhaBAD*_*-oprF-spacer-β-gluc-ΔepitopeΔβ1-ehaA* (245). In contrast to the densitometric data, the flow cytometry data indicated that the level of surface display was almost identical for all variants.

To exclude a spontaneous cell lysis, which could have tampered with the enzyme activity as determined and designated to the cell surface, the reference *P*_*rhaBAD*_*-ctxB-β-gluc-epitope-ehaA* and the four variants, *P*_*rhaBAD*_*-oprF-β-gluc-flex-ehaA* (222), *P*_*rhaBAD*_*-oprF-spacer-β-gluc-flex-ehaA* (242), *P*_*rhaBAD*_*-oprf-spacer-β-gluc-rigid-ehaA* (243), and *P*_*rhaBAD*_*-oprF-spacer-β-gluc-ΔepitopeΔβ1-ehaA* (245) were subjected to a glucose-6-phosphate (Glc-6-P) dehydrogenase assay. Glc-6-P dehydrogenase is a constitutively expressed enzyme that is released upon cell lysis. Hence, the activity of the NADP^+^ dependent Glc-6-P dehydrogenase was measured by the increase in absorbance at 340 nm due to formation of NADPH in the sample supernatants. To determine 100% Glc-6-P dehydrogenase activity, the supernatant of completely lysed host cells was measured accordingly. The ratio between the Glc-6-P dehydrogenase activity of test samples and of lysed cells was used to calculate the corresponding degree of spontaneous cell lysis in percent. All variants showed low degrees of cell lysis between 0.2 and 0.4% (Fig. 5b) at the same level of host cells (0.4%), indicating the cells remained integer after AT-FP overexpression.

### AT-FP with inversed composition for the ATB

**Fig. 6:**
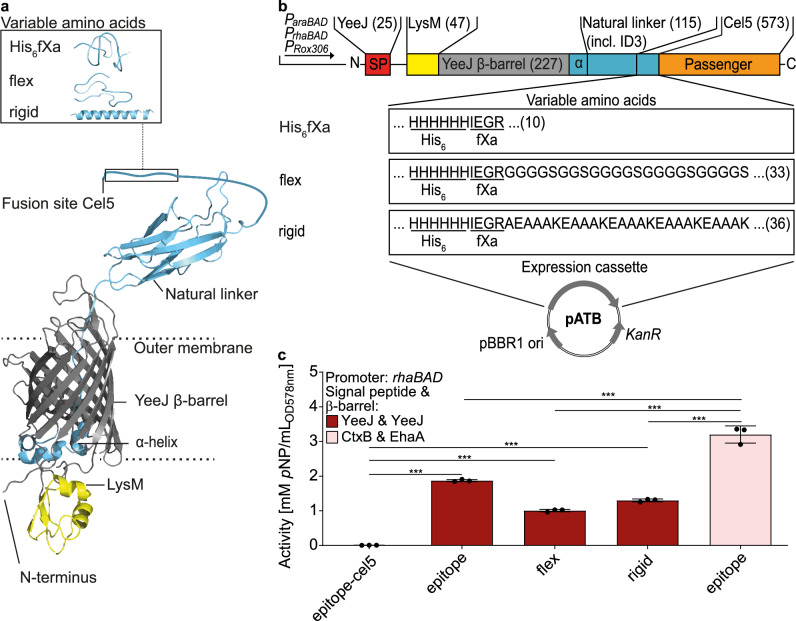
ATB AT-FP variants with inverse composition. **a** The secondary structures of the LysM, the YeeJ AT β-barrel and the natural linker were modeled with RaptorX^80^ and the variable amino acids in the linker with PEP-FOLD3^79^. **b** Inverse AT-FP contained N-terminal a YeeJ SP. LysM was followed by the YeeJ β-barrel. A linker encompassed a natural linker with ID3 and variable amino acids, including a His_6_-tag and fXa cleavage site (His_6_fXa) or His_6_fXa and G_4_S-G_2_S-(G_4_S)_3_ or His_6_fXa and A(EAAAK)_5_. The number of amino acids of the corresponding part of the AT-FP is given in brackets. All AT-FP variants are encoded by pATB. Combined with the three promoters (*P*_*araBAD*_, *P*_*rhaBAD*_, P_Rox308_), this resulted in 9 different plasmids encoding the respective variants (pATB_x51 – pATB_x53). **c** The pATB encoding AT-FP with YeeJ β-barrel under control of the *P*_*rhaBAD*_ was prepared with β-Gluc as passenger. The variants *P*_*rhaBAD*_*-yeeJ-yeeJ-epitope-β-gluc* (pATB_251*-β-gluc*), *P*_*rhaBAD*_*-yeeJ-yeeJ-flex-β-gluc* (pATB_252*-β-gluc*) and *P*_*rhaBAD*_*-yeeJ-yeeJ-rigid-β-gluc* (pATB_253*-β-gluc*), the reference *P*_*rhaBAD*_*-ctxB-β-gluc-epitope-ehaA* (pATB_211-*β-gluc*) and the control *P*_*rhaBAD*_*-ctxB-cel5-epitope-ehaA* (pATB_211-*cel5*) were tested with *p*NPG as described for the step-by-step screening (*n* = 3 biologically independent samples, ±SD, one-way ANOVA, post-test Dunnett’s multiple comparison: ***p ≤ 0.001).

In previous studies, it turned out that the surface display of certain enzymes *via* classical AT, with the passenger attached by its C-terminus to the β-barrel, led to low or even no activity. In such a case, using so-called inverse AT, with the passenger connected by its N-terminus to the β-barrel, appears to be a suitable alternative^21,54^. In consequence, variants of the inverse AT YeeJ^55^ were applied to the ATB (Fig. 6a, b). The inverse AT-FP variants comprised following domains: A N-terminal YeeJ SP, the periplasmic Lysin Motif (LysM) domain, the inverse YeeJ β-barrel, a linker encompassing the natural linker with an invasin domain 3 (ID3) and the Cel5 passenger domain at the C terminus. The natural linker and the passenger domain were artificially interspersed either by a His_6_-tag and fXa cleavage site (His_6_fXa) or by His_6_fXa and the sequence G_4_S-G_2_S-(G_4_S)_3_ (flex) or by His_6_fXa and the sequence A(EAAAK)_5_ (rigid). All inverse AT-FP variants contained the native SP of YeeJ and were listed as pATB_x51 – pATB_x53. Incorporating the variants with the inverse AT β-barrel, with *P*_*rhaBAD*_, *P*_*araBAD*_ and with *P*_*Rox306*_ expands the ATB to 81 plasmids in total (Table 1). To demonstrate the functionality of the AT-FP with inversed composition, in *P*_*rhaBAD*_*-yeeJ-yeeJ-epitope-cel5 (*pATB_251*-cel5*), *P*_*rhaBAD*_*-yeeJ-yeeJ-flex-cel5* (pATB_252*-cel5*) and *P*_*rhaBAD*_*-yeeJ-yeeJ-rigid-cel5* (pATB_253*-cel5*) the passenger Cel5 was replaced by β-Gluc and the resulting constructs were tested on enzyme activity as described for the step-by-step screening. All three variants exhibited β-Gluc activity against *p*NPG, although showing a lower activity than the reference *P*_*rhaBAD*_*-ctxB-β-gluc-epitope-ehaA* (211) (Fig. 6c). Apparently, the inversed composition of the AT-FP is not beneficial for β-Gluc. Nevertheless, pATB encoding AT-FP with YeeJ β-barrel was incorporated in the pooled plasmid library (all-in-one) or the cryopreserved strain-mix for the following ATB library screenings.

### Further applications of the ATB: bacterial laccase and human HCN2-CNBD

**Fig. 7:**
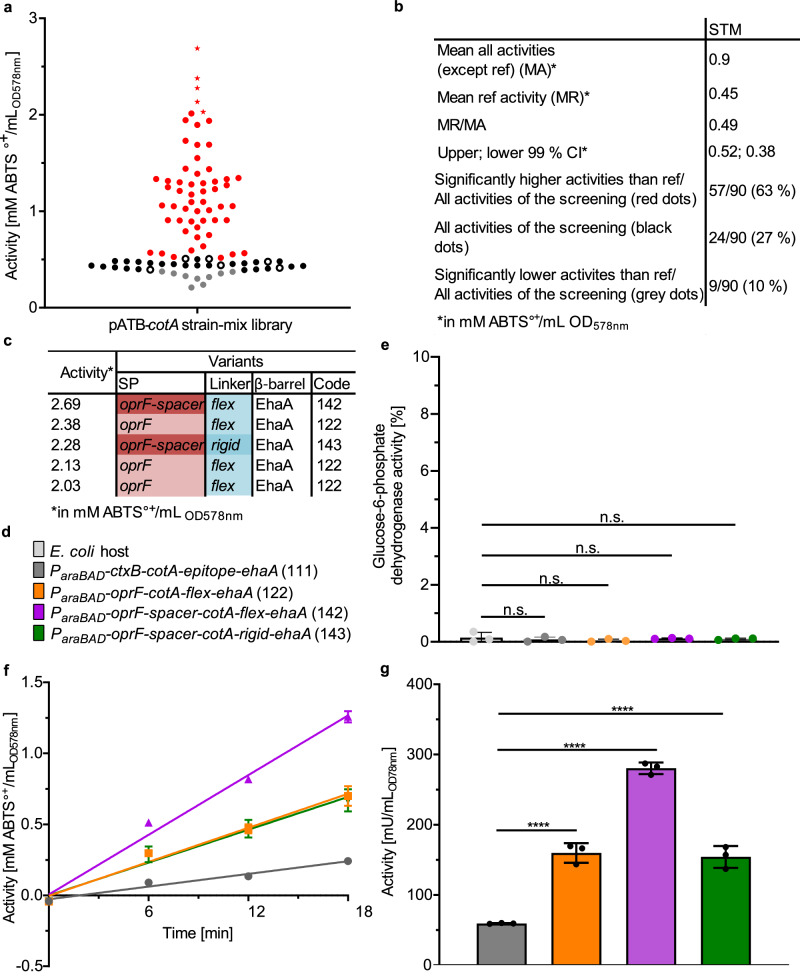
CotA activities of the ATB strain-mix library screening. **a,** **b** The pATB-*cotA* strain-mix library was prepared as described for the pATB*-β-gluc* strain-mix library but with *P*_*araBAD*_ instead of *P*_*rhaBAD*_, with *P. putida* instead of *E. coli* as host and manual induction with 0.2% L-arabinose instead of autoinduction with 2 mM L-rhamnose. In the activity assay, the substrate ABTS is converted by the laccase to ABTS^•+^, which absorbs light at a wavelength of 420 nm. The substrate conversion was determined with 20 mM ABTS in an MTP photometer at 30 °C over a period of 15 min. *P. putida* cells without a plasmid served as a blank, and variant *P*_*araBAD*_*-ctxB-cotA-epitope-ehaA* as reference. Ninety clones were screened, which corresponded to a statistical coverage of all variants of 98%. Each dot represents the activity against ABTS of either the reference *P*_*araBAD*_*-ctxB-cotA-epitope-ehaA* (111, ref.) or a variant from 90 MTP cultures. The 99% CI of the mean activity of the reference was used to identify significantly higher (red dots), equal (black dots) and lower (gray dots) activities compared to the reference (black circles). The five highest activities are marked (star). **c** The plasmids of the variants with the highest activity were subjected to sequence analysis to identify the corresponding SP, linker and β-barrel. **d** Color coding for the variants analyzed further: *E. coli* host cells, *P*_*araBAD*_*-ctxB-cotA-epitope-ehaA* (111, *P*_*araBAD*_*-oprF-cotA-flex-ehaA* (122), *P*_*araBAD*_*-oprF-spacer-cotA-flex-ehaA* (142) and *P*_*araBAD*_*-oprF-spacer-cotA-rigid-ehaA* (143). The corresponding pATB code from Table 1 is given in brackets. The color coding is kept constant in (**e**–**g**) of this figure. **e** Cell lysis of these variants, the host and the reference were assessed by measurement of Glc-6-P dehydrogenase activity in the supernatant. To determine 100% Glc-6-P dehydrogenase activity, the supernatant of completely lysed host cells was measured accordingly. The ratio between the Glc-6-P dehydrogenase activity of test samples and of lysed cells was used to calculate the corresponding degree of lysed cells in percent (*n* = 3 biologically independent samples, ±SD, one-way ANOVA, post-test Dunnett’s multiple comparison: n.s. = not significant). **f** The reference and the three variants with the highest activity from the ATB screening were verified in 200 mL scale. The activity of the cells was then determined against ABTS, and the amount of product formed was determined after 0, 6, 12, 18 min by measuring the absorbance at 420 nm. Linear regression was performed to the produced mM ABTS^•+^, for the reference (*R*^2^ = 0.97), *P*_*araBAD*_*-oprF-cotA-flex-ehaA* (122) (*R*^2^ = 0.97), *P*_*araBAD*_*-oprF-spacer-cotA-flex-ehaA* (142) (*R*^2^ = 0.99) and *P*_*araBAD*_*-oprF-spacer-cotA-rigid-*ehaA (143) (*R*^2^ = 0.95) (*n* = 3 biologically independent samples, ±SD) **g**, the slope of this linear regression from (**f**) was used to calculate the activity of the variants (*n* = 3 biologically independent samples, ±SD, one-way ANOVA, post-test Dunnett’s multiple comparison: ****p ≤ 0.0001).

First, the activity of laccase CotA from *Bacillus coagulans* that has been displayed before at the surface of *P. putida*^28^ was subjected to the strain-mix library approach for improving enzyme activity (Fig. 7). A pATB-*cotA* strain-mix library was prepared as described above, but with *P*_*araBAD*_ instead of *P*_*rhaBAD*_ and with *P. putida* as a host instead of *E. coli*. In consequence, expression was induced by 0.2% L-arabinose instead of autoinduction with 2 mM L-rhamnose. For the activity assay, 20 mM 2,2’-Azino-bis [3-ethylbenzothiazoline-6-sulfonic acid]-diammonium salt (ABTS) was used as substrate, which is converted by the laccase to ABTS•^+^, with an absorption maximum of 420 nm. An increase in absorption at 420 nm was determined for 15 min at 30 °C. *P. putida* cells without a plasmid served as a blank, and *P*_*araBAD*_*-ctxB-cotA-epitope-ehaA* (pATB_111-*cotA*) as a reference. Out of all clones obtained, 90 were analyzed on activity, statistically covering 98% of all variants theoretically possible. In Fig. 7a, each dot represents the laccase activity of either one of the 90 variants or of the reference. The 99% CI of the mean activity of the reference (black circles) was taken to identify clones with significantly higher activities (red dots, 63%), equal (black dots, 27%) and lower activities (gray dots, 10%) (Fig. 7a, b). The five best-performing variants were marked in Fig. 7a by a star, and the corresponding plasmids were subjected to sequence analysis (Fig. 7c). Variant *P*_*araBAD*_*-oprF-cotA-flex-ehaA* (pATB_122-*cotA*) appeared three times, whereas variant *P*_*araBAD*_*-oprF-spacer-cotA-rigid-ehaA* (143) (pATB_143-*cotA*) and variant *P*_*araBAD*_*-oprF-spacer-cotA-flex-ehaA* (pATB_142-*cotA*) were only found once, with variant *P*_*araBAD*_*-oprF-spacer-cotA-flex-ehaA* (142) exhibiting the highest activity.

To verify the results of the pATB library screening in a larger scale, *E. coli* host cells, *P*_*araBAD*_*-ctxB-cotA-epitope-ehaA* (111), *P*_*araBAD*_*-oprF-cotA-flex-ehaA* (122), *P*_*araBAD*_*-oprF-spacer-cotA-flex-ehaA* (142) and *P*_*araBAD*_*-oprF-spacer-cotA-rigid-ehaA* (143) were cultivated in 200 mL LB (Fig. 7d). The cell lysis of the host, the reference and the three variants *P*_*araBAD*_*-oprF-cotA-flex-ehaA* (122), *P*_*araBAD*_*-oprF-spacer-cotA-flex-ehaA* (142) and *P*_*araBAD*_*-oprF-spacer-cotA-rigid-ehaA* (143) was assessed with the Glc-6-P dehydrogenase assay as described above. All variants showed similarly low levels of approx. 0.1% cell lysis (Fig. 7e), indicating cell integrity after AT-FP overexpression.

Enzyme activity of the host, the reference and the same three variants was determined in a verification experiment (Fig. 7f, g). After linear regression, the slope of the produced mM ABTS^•+^ over time was used to calculate the activity of the variants (Fig. 7g). Variant *P*_*araBAD*_*-oprF-spacer-cotA-flex-ehaA* (142) was confirmed to be the best performer with an activity of 280 mM mU/mL_OD578nm_, representing a 4.7-fold increase in activity compared to the reference with 60 mU/mL_OD578nm_. Variant *P*_*araBAD*_*-oprF-cotA-flex-ehaA* (122) showed an activity of 160 mU/mL_OD578nm,_ and variant *P*_*araBAD*_*-oprF-spacer-cotA-rigid-ehaA* (143) showed an activity of 154 mU/mL_OD578nm_. Hence, application of the ATB strain-mix screening led to a substantial improvement of the activity of surface-displayed laccase, and indicated as well that ATB can also be applied in other Gram-negative bacteria than *E. coli*, e.g. *P. putida*.

**Fig. 8:**
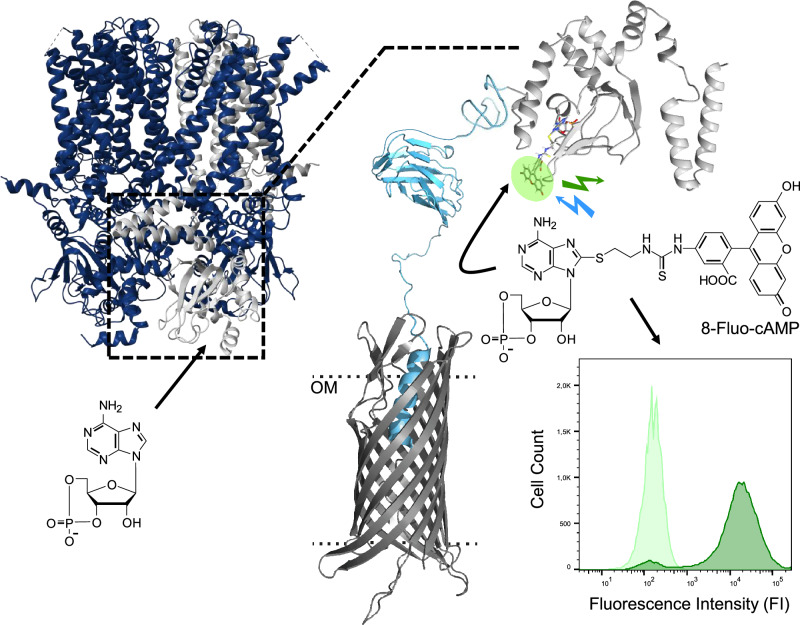
Concept of human HCN-CNBD surface display and fluorescent ligand binding. The tetrameric HCN channel and its natural ligand cyclic adenosine monophosphate (cAMP). The channel consists of four monomer subunits (light gray), each with a CNBD. The C-Linker CNBD (black box) was surface displayed using the pATB library encoding an AT-FP (β-barrel (gray), linker (light blue)). Due to the lack of a complete HCN2 structure and 91% sequence identity between the CNBD of HCN2 and HCN1, an HCN1 (PDB 5U6O) structure is shown. Binding of the fluorescent ligand 8-Fluo-cAMP to the surface-displayed CNBD can be detected as a green fluorescence signal e.g. *via* flow cytometry. The fluorescence of the bound ligand was used as a read-out during the library screening. The secondary structures of the EhaA β-barrel, the CR, and the β1 domain were modeled with RaptorX^80^ and CNBD was derived from PDB 3U10 (HCN2). 8-Fluo-cAMP was docked into the CNBD using GOLD (2023.2.0)^77^. OM = outer membrane.

As a further example, the HCN2-CNBD was chosen for surface display (Fig. 8). In recent studies, the CNBD of human HCN4 has been displayed on *E. coli* and used for drug discovery applications^56,57^. Surface-displayed HCN2-CNBD could be used for similar purposes, as well as for identifying HCN2/HCN4 selective ligands and for deciphering distinct physiological roles of both proteins. Therefore, HCN2-CNBD was subjected to the ATB strain-mix library approach to optimize surface display of HCN2-CNBD and to apply the ATB to an affinity protein, instead of an enzyme as a passenger. For screening, the well-established fluorescent ligand

8-[[2-[(Fluoresceinylthioureido)amino]ethyl]thio]adenosine-3′,5′-cyclic monophosphate (8-Fluo-cAMP) of HCN channels was used^56–60^. Consequently, an increased fluorescence of single-cell clones treated with 8-Fluo-cAMP (in comparison to control cells) would be an indication for ligand binding and hence expression of HCN2-CNBD (Fig. 8).

**Fig. 9:**
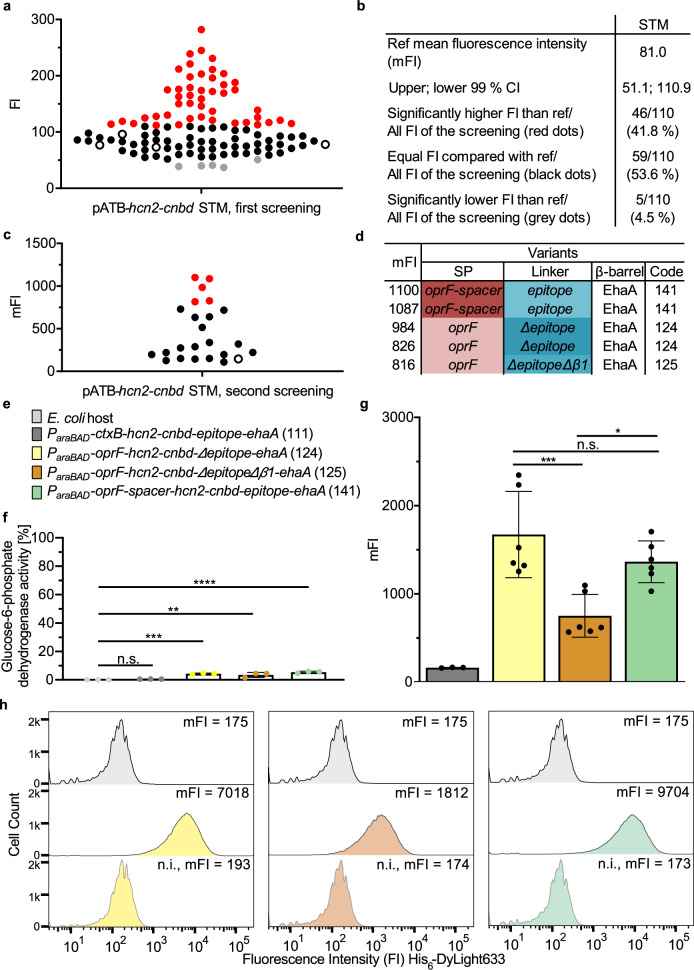
ATB strain-mix library screening for surface display of functional HCN2-CNBD. **a, b** Each dot represents the FI of 8-Fluo-cAMP bound to the surface-displayed HCN2-CNBD in MTP format. *P*_*araBAD*_*-ctxB-hcn2-cnbd-epitope-ehaA* (111) was used as reference (black circles, ref). The 99% CI of the mFI of the reference was used to identify variants with significantly higher FI ( > 99% CI, red dots) or equal FI ( = 99% CI, black dots) or lower FI ( < 99% CI) was measured. **c** In a follow-up screening, binding of 8-Fluo-cAMP to 24 variants with a significantly higher FI was confirmed in an MTP scale but using flow cytometry for the fluorescence measurement. **d** The five best-performing variants (**c**, red dots) were characterized by sequence analysis. **e** Color coding for the variants analyzed further: host, the reference and the three variants *P*_*araBAD*_*-oprF-hcn2-cnbd-Δepitope-ehaA* (124), *P*_*araBAD*_*-oprF-hcn2-cnbd-ΔepitopeΔβ1-ehaA* (125) and *P*_*araBAD*_*-oprF-spacer-hcn2-cnbd-epitope-ehaA* (141). The corresponding pATB code from Table 1 is given in brackets. Color coding is kept constant in (**f**–**h**) of this figure. **f** Lysis of cells was assessed by measurement of Glc-6-P dehydrogenase activity in the supernatant. To determine 100% Glc-6-P dehydrogenase activity, the supernatant of completely lysed host cells was measured accordingly. The ratio between the Glc-6-P dehydrogenase activity of test samples and of lysed cells was used to calculate the corresponding degree of lysed cells in percent (*n* = 3 biologically independent samples, ±SD, one-way ANOVA, post-test Dunnett’s multiple comparison: n.s. = not significant, **p ≤ 0.01, ***p ≤ 0.001, ****p ≤ 0.0001). **g** The identified variants and the reference were cultivated in a larger scale (20 mL) and binding of 8-Fluo-cAMP was measured using flow cytometry (*n* = 3 biologically independent samples, ±SD, one-way ANOVA, post-test Dunnett’s multiple comparison: reference vs. *P*_*araBAD*_*-oprF-hcn2-cnbd-ΔepitopeΔβ1-*ehaA (125) *p* ≤ 0.05, reference vs. *P*_*araBAD*_*-oprF-hcn2-cnbd-Δepitope-ehaA* (124) & *P*_*araBAD*_*-oprF-spacer-hcn2-cnbd-epitope-ehaA* (141) p ≤ 0.001; post*-*test Bonferroni’s multiple comparison of *P*_*araBAD*_*-oprF-hcn2-cnbd-Δepitope-ehaA* (124), *P*_*araBAD*_*-oprF-hcn2-cnbd-ΔepitopeΔβ1-ehaA* (125) and *P*_*araBAD*_*-oprF-spacer-hcn2-cnbd-epitope-ehaA* (141): n.s. = not significant, *p ≤ 0.05, ***p ≤ 0.001). **h** Cells were incubated with an antibody against the His_6_-tag followed by an incubation with a Dylight633-conjugated secondary antibody. The FI of the bound antibody was measured (ex. 633 nm, em. 660 nm) by flow cytometry without setting any gate. For each variant, the *E. coli* host, the induced cells expressing the AT-FP and cells harboring the respective plasmid without induction of gene expression (n.i.) were tested. The FI values of 50,000 cells per variant are shown.

The pATB*-hcn2-cnbd* strain-mix library was prepared as described above for *β-gluc*, *P*_*araBAD*_ instead of *P*_*rhaBAD*_ was used with 0.2% L-arabinose for expression induction. In a first screening, 110 pATB single-cell clones were analyzed representing a statistical coverage of all theoretically possible variants of about 98.5%. *P*_*araBAD*_*-ctxB-hcn2-cnbd-epitope-ehaA* (111) was used as a reference, since the identical combination of AT-FP components was used for HCN4-CNBD surface display in previous studies^56,57^. After growth in a 96-well MTP, variants and reference were incubated with 100 nM of 8-Fluo-cAMP for 30 min at 30 °C and fluorescence intensity (FI) was determined in a MTP photometer. A threshold was set at the 99% CI of the mFI of the reference (black circles) and variants with a significantly higher FI (red dots, 42%) or equal FI (black dots, 54%) or lower FI (gray dots, 4%) were identified (Fig. 9a, b). For a second round of screening, the 42 variants with increased FI were again cultivated in an MTP as described. Here, only 24 out of the 42 selected clones reached an OD_578nm_ = 0.5, as required for expression induction. Incubation with 8-Fluo-cAMP was analogous to before, and the FI was determined by flow cytometry (Fig. 9c).

The five variants with highest FI were subjected to sequence analysis and the SP, the linker and the β-barrel of each variant were determined (Fig. 9d). Variants *P*_*araBAD*_*-oprF-hcn2-cnbd-Δepitope-ehaA* (pATB_124-*hcn-cnbd*) and *P*_*araBAD*_*-oprF-spacer-hcn2-cnbd-epitope-ehaA* (pATB_141-*hcn2-cnbd*) were both found twice, with *P*_*araBAD*_*-oprF-spacer-hcn2-cnbd-epitope-ehaA* (141) exhibiting the highest mFI, whereas variant *P*_*araBAD*_*-oprF-hcn2-cnbd-ΔepitopeΔβ1-ehaA* (pATB_125-*hcn2-cnbd*) appeared only once.

For further analysis, the *E. coli* host, *P*_*araBAD*_*-ctxB-hcn2-cnbd-epitope-ehaA* (111) and the variants *P*_*araBAD*_*-oprF-hcn2-cnbd-Δepitope-ehaA* (124), *P*_*araBAD*_*-oprF-hcn2-cnbd-ΔepitopeΔβ1-ehaA* (125) and *P*_*araBAD*_*-oprF-spacer-hcn2-cnbd-epitope-ehaA* (141) were tested after cultivation in a 20 mL scale (Fig. 9e). The cell lysis of the reference and the three variants *P*_*araBAD*_*-oprF-hcn2-cnbd-Δepitope-ehaA* (124), *P*_*araBAD*_*-oprF-hcn2-cnbd-ΔepitopeΔβ1-ehaA* (125) and *P*_*araBAD*_*-oprF-spacer-hcn2-cnbd-epitope-ehaA* (141) was assessed with the Glc-6-P dehydrogenase assay as described above. The reference showed a cell lysis of 0.5%, while the variants *P*_*araBAD*_*-oprF-hcn2-cnbd-Δepitope-ehaA* (124), *P*_*araBAD*_*-oprF-hcn2-cnbd-ΔepitopeΔβ1-ehaA* (125) and *P*_*araBAD*_*-oprF-spacer-hcn2-cnbd-epitope-ehaA* (141) exhibited a cell lysis between 3% and 5% (Fig. 9f). The cell lysis of these variants was higher compared to variants displaying β-Gluc (Fig. 5b) or CotA (Fig. 7e), but it was in the same range as previously described for cells displaying enzymes^15^ and indicated 95 – 97% intact cells.

Binding of 100 nM 8-Fluo-cAMP was measured by flow cytometry as described above (Fig. 9g). Compared to the reference (mFI 163), variant *P*_*araBAD*_*-oprF-hcn2-cnbd-ΔepitopeΔβ1-ehaA* (125) (mFI 751) exhibited a 4.6-fold higher signal, variant *P*_*araBAD*_*-oprF-spacer-hcn2-cnbd-epitope-ehaA* (141) (mFI 1363) an 8.4-fold higher signal and variant *P*_*araBAD*_*-oprF-hcn2-cnbd-Δepitope-ehaA* (124) (mFI 1672) a 10.3-fold higher signal. The mFI of *P*_*araBAD*_*-oprF-hcn2-cnbd-Δepitope-ehaA* (124) and *P*_*araBAD*_*-oprF-spacer-hcn2-cnbd-epitope-ehaA* (141) was significantly higher than the mFI of *P*_*araBAD*_*-oprF-hcn2-cnbd-ΔepitopeΔβ1-ehaA* (125).

Finally, surface display of the HCN2-CNBD for *P*_*araBAD*_*-oprF-hcn2-cnbd-Δepitope-ehaA* (124), *P*_*araBAD*_*-oprF-hcn2-cnbd-ΔepitopeΔβ1-ehaA* (125) and *P*_*araBAD*_*-oprF-spacer-hcn2-cnbd-epitope-ehaA* (141) was confirmed by whole-cell antibody-staining using the N-terminal His_6_-tag as present in all AT-FP of the pATB library. A Dylight633-conjugated secondary antibody was used, and whole-cell fluorescence was determined again by flow cytometry (Fig. 9h). The mFI of the variants after induction of protein expression was set into relation to the mFI of cells not induced to obtain a relative mFI (Supplementary Table 3). All three variants appeared to be displayed on the bacterial cell surface, indicated by a higher mFI in comparison to cells without AT-FP gene expression. The relative mFI of 40.8 and 56.4 determined for variants *P*_*araBAD*_*-oprF-hcn2-cnbd-Δepitope-ehaA* (124) and *P*_*araBAD*_*-oprF-spacer-hcn2-cnbd-epitope-ehaA* (141), respectively, were substantially higher than the relative mFI of 10.5 determined for variant *P*_*araBAD*_*-oprF-hcn2-cnbd-ΔepitopeΔβ1-ehaA* (125), indicating that a higher level of surface display could be the reason for a higher amount of bound 8-Fluo-cAMP. Normalizing the mFI of bound 8-Fluo-cAMP by the degree of surface display as determined by antibody-staining supported this assumption (Supplementary Table 3). In this case, variant *P*_*araBAD*_*-oprF-hcn2-cnbd-ΔepitopeΔβ1-ehaA* (125), despite showing the lowest expression level, appeared to be the variant with the highest 8-Fluo-cAMP binding capacity. In summary, the pATB strain-mix library screening enabled functional HCN2-CNBD surface display and led to variants with an increased 8-Fluo-cAMP binding, with variant *P*_*araBAD*_*-oprF-hcn2-cnbd-Δepitope-ehaA* (124) and *P*_*araBAD*_*-oprF-spacer-hcn2-cnbd-epitope-ehaA* (141) appearing to be the best performers.

## Discussion

An ATB consisting of plasmids for the surface expression of a broad spectrum of recombinant proteins as a passenger in AT-FP is introduced. In its final design, the ATB consists of 81 plasmids for analyzing permutations of promotor, SP, linker and β-barrel. These can be analyzed with regard to the efficacy of the passenger’s surface display in terms of protein amount or with regard to the highest activity or ligand binding capacity of the displayed passenger. In two different library approaches, application of the ATB led to a substantial increase in the activity of surface-displayed β-Gluc and of surface-displayed laccase, as well as to a 10.3-fold increase in nucleotide binding of the human HCN2-CNBD.

For β-Gluc, it could be shown that the 4.9-fold increase in activity was not clearly due to an increase in protein amount on the cell surface (Fig. 5d, f and Supplementary Fig. 1, Supplementary Table 1, Supplementary Table 2). When the β-Gluc activity was normalized by the degree of surface display (Supplementary Table 2), it resulted in a 5.9-fold increase for the best-performing variant. It appeared that the difference between the increase in activity and level in β-Gluc surface expression was rather due to a synergistic effect of the OprF-spacer SP and the ΔepitopeΔβ1 linker. The step-by-step screening result supported this hypothesis (Fig. 4a): the activity of best-performing variant *P*_*rhaBAD*_*-oprF-spacer-β-gluc-ΔepitopeΔβ1-ehaA* (245) (12.06 mM *p*NP/mL_OD578nm_) was higher than the sum of the activities of variant *P*_*rhaBAD*_*-ctxB-β-gluc-****ΔepitopeΔβ1****-ehaA* (215, reference CtxB SP & best performer ΔepitopeΔβ1 linker, 2.33 mM *p*NP/mL_OD578nm_) and variant *P*_*rhaBAD*_*-****oprF-spacer****-β-gluc-epitope-ehaA* (241, best performer OprF-spacer SP & reference epitope linker, 3.36 mM *p*NP/mL_OD578nm_). The best-performing variant involves deletion of the epitope linker, which could indicate a limit in the generalizability of the ATB, since tags are frequently necessary for downstream experiments. However, tags do not necessarily need to appear in the epitope region. In this study, a corresponding His_6_-tag was present at the N-terminus in all mature AT-FP variants, which could be of help for downstream experiments.

As an overall trend in the step-by-step screening result, the flex linker - beside the ΔepitopeΔβ1 linker - seemed to have a beneficial effect on the activity of corresponding variants. Variants with either one of these two linkers were compared to variants with one of the remaining four linkers, but with the same SP (Supplementary Table 4). In^60^ one-to-one comparisons,^41^ exhibited no significant differences in enzymatic activities, in 11 comparisons a variant with the flex linker showed a significantly higher enzymatic activity, and in 8 comparisons this was the case for a variant with the ΔepitopeΔβ1 linker. Analyzing the influence of the SP on enzyme activity by comparing 12 variants with different SP but identical linkers, in 10 comparisons, variants with the OprF or OprF-spacer SP showed a significantly higher activity than identical variants but with CtxB or CtxB-spacer SP (Supplementary Table 5). For 2 comparisons, there was no significant difference. This could point to a beneficial effect of the OprF SP. Such a trend was not observed for the spacer when considered without SP. In 12 corresponding comparisons, 9 showed no significant difference in enzymatic activity, for 2 comparisons, variants with spacer showed a significantly higher activity (*P*_*rhaBAD*_*-oprf-β-gluc-rigid-ehaA* (223) vs. *P*_*rhaBAD*_*-oprf-spacer-β-gluc-rigid-ehaA* (243) & *P*_*rhaBAD*_*-oprF-β-gluc-ΔepitopeΔβ1-ehaA* (225) vs. *P*_*rhaBAD*_*-oprF-spacer-β-gluc-ΔepitopeΔβ1-ehaA* (245)), whereas in one comparison, the variant with spacer showed a significantly lower activity (*P*_*rhaBAD*_*-oprF-β-gluc-flex-ehaA* (222) vs. *P*_*rhaBAD*_*-oprF-spacer-β-gluc-flex-ehaA* (242)), than their identical counterparts without spacer (Supplementary Table 6). All in all, these results indicated a superior effect of the OprF SP on the activity of surface-displayed β-Gluc, whereas only a tendency for positive effects of the flex and ΔepitopeΔβ1 linker was observed. There was no clear outcome for the positive or negative influence of the spacer. Nevertheless, the results in principle indicated the suitability of the ATB for increasing the activity of cells displaying recombinant enzymes.

Generally, the activity of whole-cell biocatalysts with surface-displayed enzymes increases with the number of proteins displayed on the cell surface^23^. An optimal SP is supposed to enable efficient pre-protein translocation across the inner membrane^61–63^, which could contribute to high levels of the surface-displayed passenger. Previous studies indicated that the optimal SP is related to the protein to be exported^64,65^, but to the expression organism as well^22,66^. Since the pATB encoded EhaA β-barrel was shown to be functional for the surface display of a variety of proteins in many expression organisms beyond *E. coli* like *Pseudomonas* strains, *Ralstonia eutropha, Zymobacter palmae* and *Zymomonas mobilis*^67^, an SP adaption - not difficult to be done - could be advantageous. Nevertheless, in the present study, it was shown that the ATB-based screening for improved AT-FP variants could be performed in different *E. coli* strains, as well as in another Gram-negative host such as *P. putida*, with the best performers containing the same SP, originating from *Pseudomonas*. Hence, this could indicate that a limited number of different SP could be sufficient for an initial screening.

The linker of an AT-FP is supposed to present the passenger in the ‘sweet spot’ - the optimal combination of degree of flexibility, steric orientation, structural folding and translocation efficiency. As different linkers were shown to be optimal for different passengers^15,21,23,54^, this ‘sweet spot’ probably varies among passengers with different compositions or of different origins.

Based on these considerations, it appears reasonable that the combination of OprF-spacer SP and ΔepitopeΔβ1 linker is not the optimal combination for every passenger in each expression host. As demonstrated, the OprF-spacer SP was also optimal for CotA (Fig. 7), but not for HCN2-CNBD. Here, the two variants with the OprF SP and OprF-spacer SP showed similar results (Fig. 9). In contrast to β-Gluc, for CotA the flex linker was optimal. Likewise in contrast to β-Gluc, for HCN2-CNBD, the linkers Δepitope and epitope were optimal, both having in common native components of the EhaA AT linker (β1 and CR). Although we cannot completely exclude that general rules for optimal combinations of expression organism, SP or passenger and linker exist, such rules could not be unveiled in the present study. An optimal combination of these components remains hard to predict. This emphasizes the benefit of the ATB, providing a convenient platform for optimizing the surface display for the passenger of interest in a certain expression host.

At this point, it appears worth mentioning that the screening as applied for the ATB results in the best-performing variant with regard to a certain parameter, e.g. β-Gluc activity or 8-Fluo-cAMP binding. The increase in performance can be due to an increase in surface display or due to an altered context of the passenger at its N-terminus and its C-terminus, resulting in an improved function or even due to both. Whereas for some applications this could not be of relevance, it remains reasonable to clarify these possibilities by normalizing the value as determined for function by the degree of surface display, as it is shown in Supplementary Table 2 and Supplementary Table 3.

It should also be noted that environmental parameters such as growth medium and cultivation temperature have been reported to substantially influence surface display efficiency^23,45,46,54,68^. Although optimization of these conditions was beyond the scope of the present study, such effects could act synergistically with toolbox-based improvements and may yield even higher levels of display. In this study, different bacterial enzymes and a human nucleotide-binding protein were chosen for the ATB, indicating that passenger proteins of different functions and from different species can be subjected to its application. While our data demonstrated successful display of β-glucosidase, HCN2-CNBD, and other relatively large passengers, the mechanistic basis of how proteins of this size fold and translocate remains incompletely understood. We cannot exclude that folding limitations may restrict the upper size range of passengers, and further studies will be required to define these boundaries.

The all-in-one approach to prepare pooled plasmids for optimal surface display screening is a simple tool with a ready-to-use pATB batch. For some applications, the strain-mix approach could be of advantage, which provides the pATB-*cel5* library on demand from a single, cryo-conserved stock for applying it to the cloning of a passenger of interest. Although the ATB in this study is limited to Gram-negative bacteria, its concept and screening could be transferred to, e.g. Gram-positive bacteria, for which efficient surface display technologies exist^30,69,70^.

Library-based screening results as obtained need to be interpreted with caution due to potential biases. Quantitative assessment of variant performance with respect to linkers, spacers, and display level is recommended to support the generality of conclusions. Finally, it is possible to choose one of the 81 different plasmids, as provided in the ATB, individually, in a rational approach, e.g. based on criteria or features of the passenger for a straightforward surface display approach. Such a feature could be, e.g. an active site at the passenger’s C-terminus, which would suggest the use of a YeeJ construct to provide unrestricted access to a potential substrate. Each single plasmid of the ATB, as well as pooled libraries of pATB, can be obtained *via* Addgene (Watertown, USA).

## Methods

All key resources referenced in the following methods section are also listed in Table 2.

**Table 2:** Key resources table

Reagent/ Resource	Source	Identifier
**Chemicals, antibodies, etc**.		
1,4-Dithiothreitol	Carl Roth	Cat# 6908.1
20 mM 2,2’-Azino-bis [3-ethylbenzothiazoline-6-sulfonic acid]-diammonium salt	VWR	Cat# A1088.0005
3-((3-Cholamidopropyl)dimethylammonio)-1-propanesulfonate	Sigma-Aldrich	Cat# C3023-1g
4-Nitrophenyl-β-D-glucopyranoside	Tokyo Chemical Industry	Cat# N0235
8-Fluo-cAMP	Enzo Life Sciences	Cat# BLG-F002
Agar	Carl Roth	Cat# 5210.2
Agarose	Carl Roth	Cat# 2267.3
Alkaline Phosphatase	Carl Roth	Cat # 6024.1
Coomassie Brilliant Blue G-250	Diagonal	Cat# 1E-270.00100
CuCl_2_	Carl Roth	Cat# 2623.1
D-(+)-Arabinose	Diagonal	Cat# 5118.1
D-(+)-Glucose	Carl Roth	Cat# X997.2
DyLight 633 conjugated goat anti-mouse IgG (H + L) antibody (20 µg/mL)	Thermo Fisher Scientific	Cat# 35512
Glucose-6-phosphate	Carl Roth	Cat# 5544.1
In-Fusion Cloning	Takara Bio	Cat# 638910
Kanamycin sulfate	Carl Roth	Cat# T832.2
L-(+)-Rhamnose	Carl Roth	Cat# 4655.2
Lysogeny Broth	Carl Roth	Cat# X968.1
Monoclonal mouse anti-His_6_ antibody (10 µg/mL)	Genscript	Cat# ABIN387699
NADP^+^	Carl Roth	Cat# AE13.1
Oligonucleotides (listed in Supplementary Table 7)	Sigma-Aldrich	customized
Phenylmethylsulfonyl fluoride	Carl Roth	Cat# 6367.1
Proteinase K	Diagonal	Cat# A3830,0100
T4 DNA Ligase	Thermo Fisher Scientific	Cat# EL0011
VeraSeq High Fidelity DNA Polymerase	Biozym	Cat# 280390
XhoI/ KpnI/ BglII/	Thermo Fisher Scientific	Cat # FD0694/ FD0524/ FD0084
**Bacterial strains**		
*E. coli* UT5600	^76^	
*P. putida* KT2440	Thermo Fisher Scientific	Strain Code: ATCC 47054
**Commercial kits**		
NucleoSpin Kit	Th. Geyer	Cat# 740609250
Roti Prep Plasmid Mini-XL	Carl Roth	Cat# 8546.3
**Services**		
Sequence analysis	Microsynth AG	Economy Run
Synthesis of string DNA	Thermo Fisher Scientific (*β-gluc, hcn2-cnbd*) Twist Bioscience HQ (*cotA, hcn2-cnbd*)	customized
**Software and algorithms**		
Adobe Illustrator Artwork 16.0	Adobe Inc.	
ChimeraX V1.6.1	^71^	
DeepL	DeepL SE	
FACS Diva 8.0	BD Life Sciences	
FlowJo 10.8.1	BD Life Sciences	
GLUE-IT CASTER 2.0	^53^	
GOLD (2023.2.0 (Build 382240)	^77^	
GraphPad Prism V6	GraphPad Software	
Image J 1.50i	^78^	
NEBiocalculator® version 1.15.0	New England Biolabs	
PEP-FOLD3	^79^	
RaptorX	^80^	

### Computational methods

The secondary structures of the EhaA AT β-barrel, the EhaA CR, the EhaA β1 domain, the LysM, the YeeJ β-barrel and the natural linker were modeled with RaptorX. Variable amino acids in the linker (epitope, flex, rigid) were modeled with PEP-FOLD3. Image J 1.50i was used to perform the densitometric analysis of the SDS-PAGE gel. NEBiocalculator® version 1.15.0 was used for calculations regarding ligation reactions in pATB*-β-gluc* step-by-step and all-in-one library preparation. GLUE-IT CASTER 2.0 was used to calculate the statistical coverage of the 24 pATB*-β-gluc* variants for the screening of the pATB*-β-gluc* all-in-one and strain-mix library. Data obtained from library screenings and high-performing variant verification were analyzed and visualized with GraphPad Prism V6. Visualization of Figs. 1, 2a, b, and 3 were performed with Adobe Illustrator Artwork 16. Analysis of flow cytometry data was done with FlowJo 10.8.1. The fluorescent ligand 8-Fluo-cAMP was docked into PDB 3U10 using GOLD 2023.2.0 (Build 382240), and corresponding protein structures were visualized with ChimeraX V1.6.1^71^. During the preparation of this manuscript, the large language model DeepL SE was used to improve the clarity and readability of the text. All content was carefully reviewed and verified after the use of the large language model.

### Bacterial cultivation conditions

*Escherichia coli* UT5600 is a K12 derivative which is OmpT negative (F¯, ara-14, leuB6, azi-6, lacY1, proC14, tsx-67, entA403, trpE38, rfbD1, rpsL109, xyl-5, mtl-1, thi-1, Δ(ompT-fepC)266)). Thereby, OmpT-dependent release of the passenger is prevented. *E. coli* UT5600 has been successfully applied in many studies on bacterial surface display before^5,34,54,72^. *Escherichia coli* strains DH5α and UT5600 were cultivated in 1/5 filled 100 mL or 1 L shake flasks containing LB medium (37 °C, 200 rpm) or on LB agar plates (37°C), both containing Kan50, if selection was desired. Cultivations of *E. coli* pATB*-β-gluc* strains in 1 L shake flasks were inoculated with 1% (v/v) from the overnight culture. When the cell suspension reached an optical density at 578 nm OD_578nm_ of 0.5, AT-FP gene expression was induced *via* the addition of 2 mM L-(+)-rhamnose (final concentration) and the cultivation was prolonged for an additional 2 h. *E. coli* pATB*-β-gluc* strains were also cultivated in 96-well MTPs. Inner wells contained the cells which were cultivated on a 96-well MTP shaker (25 °C, 20 h, 600 rpm) in 200 µl LB Kan50 medium, 0.1% (w/v) glucose and 2 mM L-(+)-rhamnose allowing autoinduction of AT-FP gene expression. Wells at the outer edge were filled with sterile ddH_2_O to prevent evaporation effects.

### Plasmid construction

In the entire *Methods* section, the three-digit code (x = promoter, y = SP ± spacer, z = linker) to denote pATB is used (Table 1). In a first step, the pATB 111, 121, 131, 141, 211, 212, 213, 214, 215, 216, 221, 231, 241, 311, 321, 331, 341 were constructed. Therefore, the DNA sequence encoding Cel5 from *Hahella chejuensis* (Uniprot ID: Q2SFD8) was synthesized as a string of DNA including 3’ *kpnI* and 5’ *xhoI* restriction sites (Thermo Fisher Scientific). The string DNA as used was codon optimized for *E. coli,* and the final DNA sequence is given in Supplementary Fig. 3. Restriction/ligation *via* XhoI/KpnI and T4 DNA ligase was used to exchange the passenger DNA sequence in previously published p*P*_*BAD*_-MATE-*CsbglA*^27^, leading to pATB_111 (*P*_*araBAD*_, *CtxB, epitope*), in p*oprFSP*-MATE-*estA*^22^, leading to p*oprFSP*-MATE-*cel5* (*P*_*rhaBAD*_, *CtxB, epitope*) and in *P*_*Rox306*_-MATE-*CsbglA*^27^, leading to pATB_311 (*P*_*Rox306*_*, CtxB, epitope*). The following plasmids were generated using In-Fusion Cloning as described by the manufacturer. Therefore, DNA sequences of the respective template plasmids were amplified *via* PCR. The designations of the primers that were used for PCR are given in brackets (fw = forward, rv = reverse), and respective sequences are summarized in Supplementary Table 7. p*oprFSP*-MATE-*cel5* (774 (fw) & 770 (rv)) was used as template plasmid to generate p*oprFSP*-MATE-*cel5* (= pATB_121 (*P*_*araBAD*_, *OprF, epitope*)) containing the myc-tag encoding sequence in the linker. pATB_111 (568 (fw) & 984 (rev)) and previously published pPQ28^15^ (931 (fw) & 534 (rv)) served as template plasmids to generate pATB_211 (*P*_*rhaBAD*_*, CtxB, epitope*). pATB_121 (1584 (fw) & 984 (rv)) and pATB_211 (931 (fw) & 1568 (rev)) were used as template plasmids to generate pATB_221 (*P*_*rhaBAD*_*, OprF, epitope*). pATB_121 (1597 (fw) & 984 (rv) and pATB_311 (932 (fw) & 915 (rv)) were the template plasmids to generate pATB_321 (*P*_*Rox306*_*, OprF, epitope*). pATB_111, 121, 211, 221, 311 and 321 (3289 (fw) & 3290 (rv)) were used as template plasmids in individual reactions, to generate pATB_131, 141, 231, 241, 331, and 341, which differed from their template plasmids by the spacer DNA sequence in the pro-region coding DNA. pATB_211 was used as a template in three reactions (878 (fw) & 879 (rv), 883 (fw) & 880 (rev), 814 (fw) & 882 (rev) respectively), to construct the deletion variants encoding pATB_214 (*P*_*rhaBAD*_, *CtxB, Δepitope)*, pATB_215 (*P*_*rhaBAD*_, *CtxB, ΔepitopeΔβ1)*, pATB_216 (*P*_*rhaBAD*_, *CtxB, ΔepitopeΔβ1ΔCR)*. pATB_214 was the template in two reactions (3291 (fw) & 3293 (rev), 2858 (fw) & 2857 (rev)) to generate pATB_212 (*P*_*rhaBAD*_, *CtxB, flex)* and pATB_213 (*P*_*rhaBAD*_, *CtxB, rigid)*.

In a second step, the pATBs 112–116, 122–126, 132–136, 141–146, 22–226, 232–236, 242–246, 312–316, 322–326, 332–336, 342–346 were generated based on the previously constructed pATBs using restriction/ligation *via* XhoI/KpnI/BglII (10 U/enzyme, 37 °C, 2 h) and T4 DNA ligase (5 U, 4 °C, overnight). pATB_111, 121, 131, 141, 211, 221, 231, 241, 311, 321, 331, and 341 were digested with BglII/XhoI to obtain DNA backbones that individually contained each possible combination of promoter DNA (*P*_*araBAD*_, *P*_*rhaBAD*_, *P*_*Rox306*_) and SP encoding DNA (*CtxB*, *OprF*, *CtxB-spacer*, *OprF-spacer*). pATB_211, 212, 213, 214, 215 and 216 were digested with KpnI/BglII (10 U/enzyme, 37 °C, 2 h) to generate DNA inserts that individually encoded the linker variants (*epitope*, *flex*, *rigid*, *Δepitope*, *ΔepitopeΔβ1*, *ΔepitopeΔβ1ΔCR*). The respective DNA backbone sequences were ligated with respective DNA insert sequences. To produce the plasmids, respective ligation or In-Fusion mixtures were used to transform heat-shock competent *E. coli* DH5α cells. The correctness of the plasmids was validated *via* colony PCR (primers listed in Supplementary Table 7) and sequence analysis.

In a third step, the pATBs 151-153, 251-253, 351-353 were generated. The passenger DNA sequence of previously published pDG11-*estA*^54^ (*P*_*araBAD*_*, YeeJ, epitope)* was changed to *CsbglA via* the described restriction and ligation approach using p*P*_*BAD*_-MATE-*CsbglA*^27^, XhoI/KpnI and T4 DNA ligase. pDG11-*CsbglA*^54^ was used as template in two PCR reactions ((I) 3676 (fw) & 3572 (rev); (II) 1815 (fw) & 2532 (rev)). pATB_212 ((I) 1230 (fw) & 2071 (rv)) and pATB_213 ((II) 2537 (fw) & 3573 (rv)) were used as template plasmids in individual PCR reactions. The respective DNA fragments were used for In-Fusion Cloning to generate (I) pATB_152-*CsbglA* (*P*_*araBAD*_*, YeeJ, flex*) and (II) pATB_153-*CsbglA* (*P*_*araBAD*_*, YeeJ, flex*). Subsequently, the promoter sequences of pDG11-*estA*^54^, pATB_152-*CsbglA* and pATB_153-*CsbglA* were changed from *P*_*araBAD*_ to *P*_*rhaBAD*_ and *P*_*Rox306,*_ respectively, via PCR and In-Fusion Cloning. pDG11-*estA*^54^, pATB_152-*CsbglA* and pATB_153-*CsbglA* (3569 (fw) & 3570 (rv)) and pATB_211 (931 (fw) & 3544 (rev)) were used as template plasmids to generate pATB_251 (*P*_*rhaBAD*_*, YeeJ, epitope*), pATB_252 (*P*_*rhaBAD*_*, YeeJ, flex*), pATB_253 (*P*_*rhaBAD*_*, YeeJ, rigid*). pDG11-*estA*^54^, pATB_152-*CsbglA* and pATB_153-*CsbglA* (2531 (fw) & 3570 (rv)) and pATB_311 (931 (fw) & 915 (rev)) were used as template plasmids to generate pATB_351 (*P*_*Rox306*_*, YeeJ, epitope*), pATB_252 (*P*_*Rox306*_*, YeeJ, flex*), pATB_253 (*P*_*Rox306*_*, YeeJ, rigid*). The passenger DNA sequences of pATB 151-153, 251-253, 351-353 were changed to *cel5 via* the described restriction and ligation approach using pATB_211, XhoI/KpnI and T4 DNA ligase. The correctness of the plasmids was validated via colony PCR (primers listed in Supplementary Table 7) and sequence analysis.

### pATB*-β-gluc* step-by-step, all-in-one and strain-mix library preparation

Three different approaches were applied to prepare libraries with the pATB for the subsequent screening. In a step-by-step approach, approx. 50 fmol ( = approx 300 ng) DNA of each of the 24 pATB containing *P*_*rhaBAD*_ and EhaA β-barrel coding DNA (pATB-*cel5*, pATB_211 – pATB_246) was treated in a separate reaction with XhoI/KpnI (10 U/enzyme) and FastAP (2 U, 37 °C, 2 h). Likewise, approx. 367.1 fmol ( = approx 2 µg) DNA of p*β-gluc* previously published as pBBR1MCS-2_BAD_-*Cs*BglA-LYTH carrying the *β-gluc* DNA sequence (Uniprot ID: P10482) was digested with XhoI/KpnI but without FastAP. The DNA fragments were separated by agarose gel electrophoresis (1% agarose (w/v), 120 V, 45–80 min), and pATB DNA backbones and *β-gluc* DNA inserts were purified *via* silica membrane using spin columns. For the separate ligation reactions, the molar insert: backbone ratio was supposed to be 3:1. Thus, approx. 2 fmol DNA/pATB backbone ( = approx 10 ng considering an average backbone size of 8152 bp) and approx. 6 fmol of the *β-gluc* DNA insert ( = 5 ng considering an insert size of 1366 bp) was required for the ligation reaction catalyzed by T4 DNA ligase (5 U, 4 °C, overnight) (NEBioCalculator® version 1.15.0). Heat shock competent *E. coli* DH5α cells were separately transformed with 5 µL of the ligation mixture to produce the collection of 24 plasmids termed pATB *P*_*rhaBAD*_*-β-gluc* step-by-step library. Correct plasmids (validation as described) were used to separately transform heat-shock competent *E. coli* UT5600 cells.

In an all-in-one approach, the 24 pATB_211 – pATB_246 were manually pooled in equimolar concentrations (approx. 3.4 fmol DNA/pATB = approx. 20 ng DNA/pATB). The pooled pATB (approx. 81.6 fmol = approx 500 ng pATB DNA) were subjected to restriction digest using XhoI/KpnI (10 U/enzyme, 37 °C, 2 h) and added FastAP (2U) was supposed to prevent religation of emerging DNA fragments. Approx. 367.1 fmol ( = approx 2 µg) DNA of p*β-gluc* carrying the *β-gluc* DNA sequence was also digested with XhoI/KpnI but without FastAP. The 24 pATB DNA backbones and the *β-gluc* DNA insert were separated from the remaining plasmid DNA *via* agarose gel electrophoresis (inserts: 1% agarose (w/v), 120 V, 45 min; backbones: 1% agarose (w/v), 120 V, 80 min) with subsequent silica-membrane based purification using a spin column. For the ligation reaction, the molar insert: backbone ratio was supposed to be 3:1. Thus, 12 fmol backbone DNA in total ( = approx 60 ng DNA considering an average backbone size of 8152 bp) and approx. 36 fmol ( = approx. 30 ng DNA considering an insert size of 1366 bp) of the *β-gluc* DNA insert was required for the ligation reaction catalyzed by T4 DNA ligase (5 U, 4 °C, overnight) (Calculations: NEBioCalculator® version 1.15.0). Ligation reaction resulted in the pATB*-β-gluc* all-in-one library. 5 µL of the ligation mixture was used to transform heat shock competent *E. coli* UT5600 cells, and cells were plated out on two plates to increase single-cell clone library size.

In a strain-mix approach, the mixture of the 24 manually pooled pATB-*cel5* was used to transform heat-shock competent *E. coli* DH5α. The cells were cultivated on LB Kan50 agar plates. The cells were detached from the plates using 5 mL LB Kan50 medium. This cell suspension was mixed with glycerol (25% (v/v) final), and the cells were preserved cryologically in 0.5 mL stocks. An entire stock was used to inoculate an overnight culture. The plasmids harbored by cells from the overnight culture were isolated, resulting in a pooled pATB*-cel5* strain-mix library, which was further treated as described in the protocol for the all-in-one approach.

### β-Gluc library screening and verification

Cells were either picked from four randomly chosen clones per variant of *E. coli* UT5600 cells transformed with the pATB*-β-gluc* step-by-step library or from 105 randomly chosen clones of *E. coli* UT5600 cells transformed with the pATB*-β-gluc* all-in-one or strain-mix library. The strains were individually cultivated in a well of an MTP as described and subsequently subjected to an activity test in MTP format. The MTPs containing cultivated cells were centrifuged (3900 × *g*, 4 °C, 15 min) and 200 µl 0.1 M Na-citrate buffer (pH 6) was used to wash twice and resuspend the cells. The cell suspensions were diluted 1:10 with 0.1 M Na-citrate buffer (pH 6) in a fresh MTP, and the OD_578nm_ was measured in a MTP reader. 30 µl of the diluted cell suspension was transferred to a fresh plate, and the activity test was started by adding 30 µl of 10 mM preheated (55 °C, 3 min) *p*NPG (final concentration 5 mM). The MTP was incubated (55 °C, 9 min) and centrifuged (3900 x *g*, 4 °C, 3 min). To quantify *p*NP, 25 μL supernatant of each sample was transferred to a fresh MTP, diluted with 25 μL 0.1 M sodium citrate buffer (pH 6), and mixed with 50 μL of 2 M Na_2_CO_3_. The absorption was photometrically determined at 405 nm, adjusted for the absorption of equally treated *E. coli* UT5600 host cells, normalized to the previously determined OD_578nm_ and used to calculate activities with a calibration curve (*y* = 3,086x + 0,0053). The sequence of the plasmids harbored by high-performing variants of the all-in-one or strain-mix approach was analyzed. To verify high-performing variants that were identified *via* pATB library screening in a MTP scale, respective variants were cultivated in a 1 L shake flask scale as described in previous section, harvested (3900 × *g*, 4 °C, 15 min), washed with 0.1 M sodium citrate buffer (pH 6) and subjected to an activity test. Therefore, 15 samples were prepared. Per reaction tube, 30 μL of the cell suspension OD_578nm_ 0.2 (final OD_578nm_ of 0.1) were mixed with 30 μL of 10 mM preheated (55 °C, 3 min) *p*NPG (final concentration 5 mM) to start the reaction. The mixtures incubated (55 °C, 900 rpm) and the reaction was stopped after 0, 3, 6, and 9 min by placing the reaction tubes on ice. The reaction tubes were centrifuged (3900 × *g*, 4 °C, 3 min) and the supernatant of each sample was transferred to a fresh MTP. Subsequently the samples were processed as described for *p*NP quantification. *p*NP concentrations determined for 0, 3, 6, and 9 min reaction time were used to determine the activity of the cells. The activity was normalized to 1 mL of a cell suspension with an OD_578nm_ of 1 (mU/(mL_OD578nm_)).

### Membrane protein isolation and protease accessibility of β-Gluc variants

Membrane protein isolation (MPI) was performed as described previously^73^. Cells from 40 ml culture broth were pelleted (10 min, 3850 × *g*, 4 °C). Prior to MPI, the surface accessibility of β-Gluc was tested by applying proteinase K (prot. K). Therefore, the cells were resuspended in 1 ml 0.1 M PBS and incubated after addition of 250 µg prot. K for 1 h at 37 °C. Protease digest was stopped by adding 50 µl of 0.1 M Phenylmethylsulfonyl fluoride. The mixture was centrifuged (5 min, 3850 × *g*, 4 °C), the cells were resuspended in 1.5 ml 0.2 M Tris-HCl buffer (pH 8) and subsequently treated as described for MPI. For SDS-PAGE analysis, the samples were boiled for 20 min at 95 °C in SDS-PAGE sample buffer containing 30 mM DTT and separated in a 12.5% polyacrylamide gel. Coomassie Brilliant Blue G-250 was used to stain the proteins in the gel.

### Flow cytometry analysis of β-Gluc variants

To test surface exposure of the β-Gluc, cells were analyzed by flow cytometry after treatment with fluorescence labeled antibodies. Cultivations of *E. coli* pATB*-β-gluc* strains in 100 mL shake flasks were inoculated with 1% (v/v) from the overnight culture. When the cell suspension reached an OD_578nm_ of 0.5, AT-FP gene expression was induced *via* addition of 2 mM L-(+)-rhamnose (final concentration) and the cultivation prolonged for additional 2 h. After harvest, cells were washed three times with filter-sterilized PBS. 1 mL of cells (OD_578nm_ 0.2) were treated with 10 µg/mL monoclonal mouse Anti-His_6_ antibody for 16 h. Cells were washed three times with PBS and then treated with 20 µg/mL secondary DyLight 633 conjugated goat anti-mouse IgG (H + L) antibody in the dark for 1 h. After a final round of washing, the fluorescence of 50,000 cells was analyzed by flow cytometry (ex. 633 nm, em. 660 nm).

### CotA library preparation, screening and verification

The DNA sequence of *Bacillus coagulans* laccase *cotA* was synthetized in an *E. coli* codon optimized version. The pATB*-cotA* strain-mix library was prepared as described for the pATB*-β-gluc* strain-mix library but with pooled pATB with *P*_*araBAD*_ instead of *P*_*rhaBAD*_. *P. putida* KT2440 cells were transformed with this pATB*-cotA* library and cultured in 96-well MTP wells with 200 µL LB medium, Kan50, 0.4 mM CuCl_2_ for 6.5 h (30 °C, 600 rpm) followed by addition of 0.2% L-arabinose and static incubation (18 h, 30 °C). The cells were washed three times with 0.1 M Na-acetate buffer (pH 5.6) and the OD_578nm_ was determined. The substrate ABTS is converted by the laccase to ABTS^•+^ (*ε* = 36.000 M^−1^ cm^−1^^74^), which absorbs light at a wavelength of 420 nm. The activity was determined with 20 mM in an MTP photometer at 30 °C over a period of 15 min under constant shaking. The slope of the increase in absorbance within the linear range was calculated. The calculated slope was normalized to the OD_578nm_ of the cell suspension in the respective cavity. As a reference, *P. putida* pATB_111*-cotA* cells were cultured in six 96-well MTP wells, and their activity was tested. As a blank, *P. putida* cells without a plasmid were cultivated in three wells, and their activity was tested. Ninety clones were screened. The plasmids of the five highest performing variants were subjected to sequence analysis to identify the corresponding signal SP, linker an β-barrel. The five variants with the highest activity from the toolbox screening were verified on a 200 mL scale. Cells were cultured in 200 mL LB medium containing 1 mM CuCl_2_ at 30 °C and 200 rpm until an OD_578nm_ of 0.9 was reached. The cultures were then transferred to centrifuge tubes, and optionally 0.2% (w/v) L-arabinose was added to induce gene expression and the cells incubated statically for further 18 h. The activity of the cells was then determined in 2 mL reaction tubes. Reaction mixtures contained 100 µL of 60 mM ABTS and 100 µL of the respective cell suspension with an OD_578nm_ of 0.5 in 0.1 M Na-acetate buffer (pH 5.6). The preparations were incubated at 30 °C, 1000 rpm and the amount of product formed was determined after 0, 6, 12, 18 min by measuring the absorbance at 420 nm. Linear regression was performed on the produced mM ABTS^•+^. The slope of this linear regression was normalized to the OD_578nm_ = 1 of the cell suspension and was used to calculate the activity of the variants in mU/mL_OD578nm_

### HCN2-CNBD library preparation, screening and verification

The DNA sequence of the C-Linker and CNBD of HCN2 was synthetized in an *E. coli* codon-optimized version. The pATB*-hcn2-cnbd* strain-mix library was prepared as described for the pATB*-β-gluc* strain-mix library but with pooled pATB with *P*_*araBAD*_ instead of *P*_*rhaBAD*_. For a first screening round, *E. coli* cells were transformed with this pATB-*hcn2-cnbd*-library and cultured in 96-well MTP wells until an OD_578nm_ of 0.5 (37 °C, 200 rpm), followed by addition of 0.2% L-arabinose and an additional incubation for 2 h at 23 °C (200 rpm). As a reference, *E. coli* pATB_111-*hcn2-cnbd* was included in 4 wells. The cells were washed three times with phosphate-buffered saline (PBS, pH 7.4), adjusted to an OD_578nm_ of 0.35 and resuspended in 90 µL PBS with the addition of 0.1% 3-((3-Cholamidopropyl)dimethylammonio)-1-propanesulfonate (CHAPS). 10 µL of a 1 µM 8-Fluo-cAMP solution were added and incubated at 30 °C, 600 rpm for 30 min. Following the incubation, cells were washed twice and resuspended in 100 µL PBS. The FI of bound 8-Fluo-cAMP was measured with an MTP photometer (ex. 488 nm, em. 525 nm). In the first screening 110 clones were screened. For the second screening 24 variants with significantly higher FI compared to the reference HCN2-CNBD and fast growth in liquid culture were tested again. The cells were cultured and incubated with 8-Fluo-cAMP as before, but the FI of the bound ligand was measured by flow cytometry (ex. 488 nm, em. 530 nm). Here, the single-cell fluorescence of 50,000 cells per variant was recorded. The plasmids of the five clones with the highest mFI were subjected to sequence analysis to identify the corresponding SP, linker and β-barrel. The identified variants were then verified in a 20 mL scale. Cells were cultured in 20 mL LB medium until an OD_578nm_ of 0.5 was reached (37 °C, 200 rpm). Gene expression was induced with the addition of 0.2% L-arabinose and the cells were incubated for another 2 h at 23 °C (200 rpm). Following three washing steps in PBS, OD_578nm_ was adjusted to 0.35 in 1 mL particle-free PBS, and after another washing step, cells were resuspended in 90 µL PBS + 0.1% CHAPS. 10 µL of a 1 µM 8-Fluo-cAMP solution were added and the samples were incubated for 30 min at 30 °C (600 rpm). The cells were washed once before the FI of bound 8-Fluo-cAMP of 50,000 cells per variant was measured by flow cytometry (ex. 488 nm, em. 530 nm). The FI of 8-Fluo-cAMP bound to pATB_111-HCN2-CNBD was measured as a reference. To confirm the surface display of HCN2-CNBD, antibody labeling against the His_6_-tag was performed. *E. coli* BL21 pATB124*-hcn2-cnbd*, pATB125*-hcn2-cnbd* and pATB141*-hcn2-cnbd* were cultured in 20 mL LB medium respectively (37 °C, 200 rpm, until OD_578nm_ of 0.5). Gene expression was induced with 0.2% L-arabinose at 23 °C for 2 h (200 rpm). After harvest, cells were washed three times with PBS. 1 mL cell suspension (OD_578nm_ 0.35) was sedimented and resuspended in 100 µL particle-free PBS and treated with 10 µg/mL monoclonal mouse Anti-His_6_ antibody for 1 h at 23 °C. Cells were washed three times with PBS and then treated with 20 µg/mL secondary DyLight633-conjugated goat anti-mouse IgG (H + L) antibody for 1 h (dark, 23 °C). Following one washing step with particle-free PBS, the fluorescence of the bound secondary antibody was measured using flow cytometry (ex. 633 nm, em. 660 nm).

### Glc-6-P dehydrogenase activity test

To evaluate cell lysis, Glc-6-P dehydrogenase activity was measured. The enzyme is constitutively expressed in the cytosol and found in the supernatant only upon cell lysis. Washed cells displaying β-Gluc were resuspended in 0.1 M sodium citrate buffer (pH 6), cells displaying CotA were resuspended in 0.1 M Na-acetate buffer (pH 5.6), and cells displaying HCN2-CNBD were resuspended in PBS + 0.1% CHAPS. The OD_578nm_ was set to 1 and then centrifuged for 5 min at 10000 g. 90 μL supernatant were mixed with 10 μL of a reaction solution (40 mM Glc-6-P, 20 mM NADP^+^). Formation of NADPH was measured at 340 nm for up to 1 h in a 96-well plate at 30 °C. As a reference, host cells in respective buffer with an OD_578nm_ of 1 were lysed through sonification, centrifuged for 5 min at 10,000 × g and the supernatant was analyzed as described above.

### Statistics and Reproducibility

The activity of all β-Gluc variants in the pATB step-by-step screening was determined as *n* = 4 biologically independent samples. The activity of β-Gluc variants with inverted composition, the verification of the highest performing variants of β-Gluc, CotA and HCN2-CNBD and the assessments of cell lysis were determined as *n* = 3 biologically independent samples. Each biological independent sample started with a bacterial colony picked from an agar plate. In the pATB-*β-gluc* all-in-one and strain-mix library screening, the activity of 105 variants was tested. In the pATB-*cotA* strain-mix library screening 90 variants were tested, and in the pATB-*hcn2-cnbd* strain-mix library screening, 110 (first round) and 24 (second round) were tested. Bacterial colonies were the result of a transformation with respective plasmids. A K-S test was performed with the cumulative frequency distributions of the pATB-*β-gluc* all-in-one and pATB-*β-gluc* strain-mix screening data sets. FACS Diva 8.0 was used for collecting the flow cytometry data with FACS Aria III device (Becton Dickinson, Heidelberg). To measure the Dylight633 fluorescence, an excitation wavelength of 633 nm and emission wavelength filters of 660/20 nm (band-pass) were used. GFP fluorescence was excited at 488 nm and measured with an emission wavelength filter of 530/30 nm (band-pass) and 502 nm (long-pass). No gating strategy was applied. FlowJo version 10.8.1 was used for analyzing the flow cytometry data. Surface accessibility of β-Gluc and HCN2-CNBD was determined by flow cytometry analysis measuring 50,000 cells per variant. Sequence analysis of high-performing variants was done once per sequence of interest for each variant.

Statistical analyses were done with Microsoft Excel 2024 and GraphPad Prism 6 (GraphPad Software Inc.). Results are presented as mean ± SD as described in the figure legends. To test statistical significance, two-tailed Student’s *t* test or two-tailed one-way ANOVA with, where appropriate, either Dunnett’s or Bonferroni multiple comparison post-test was done. Different levels of significance (p-values) are indicated by asterisks (n.s. = not significant, *p  <  0.05, **p  <  0.01, ***p  <  0.001, ****p  <  0.0001). GLUE-IT CASTER 2.0 was used to calculate the statistical coverage of the pATB variants for the screening of the pATB all-in-one and strain-mix library.

### Reporting summary

Further information on research design is available in the Nature Portfolio Reporting Summary linked to this article.

## Supplementary information

Supplementary Information

Reporting Summary

## Data Availability

Numerical source data for graphs and charts can be found on Figshare following the 10.6084/m9.figshare.26969659^75^. All other data are available from the corresponding author J.J. (joachim.jose@uni-muenster.de) on reasonable request. Data in this manuscript and its supplementary information are presented in aggregated form wherever possible. Single plasmids of the ATB, as well as pooled plasmid libraries can be obtained from Addgene (Watertown, USA).

## Code availability

No custom code or mathematical algorithm that is deemed central to the conclusions was used in this study.
