# Supplementary material for: A plasmid toolbox for the easy autodisplay of recombinant proteins and its optimization
Source: Commun Biol. 2026 May 21;9:694. doi: 10.1038/s42003-026-10324-7 (PMC13195121; doi:10.1038/s42003-026-10324-7)
Supplement: Supplementary file 1 — Supplementary Information [file 42003_2026_10324_MOESM1_ESM.pdf]

Supplementary information

A plasmid toolbox for the easy autodisplay of recombinant proteins and its optimization.

Christoph Furtmann*, Philip Röhe*, Katrin Gesing, Hanna Kuss, Florian Lenz & Joachim Jose.

University of Münster, Institute for Pharmaceutical & Medicinal Chemistry, PharmaCampus, Münster, Germany

*These authors contributed equally: Christoph Furtmann, Philip Röhe.
Corresponding author: Joachim Jose (joachim.jose@uni-muenster.de)

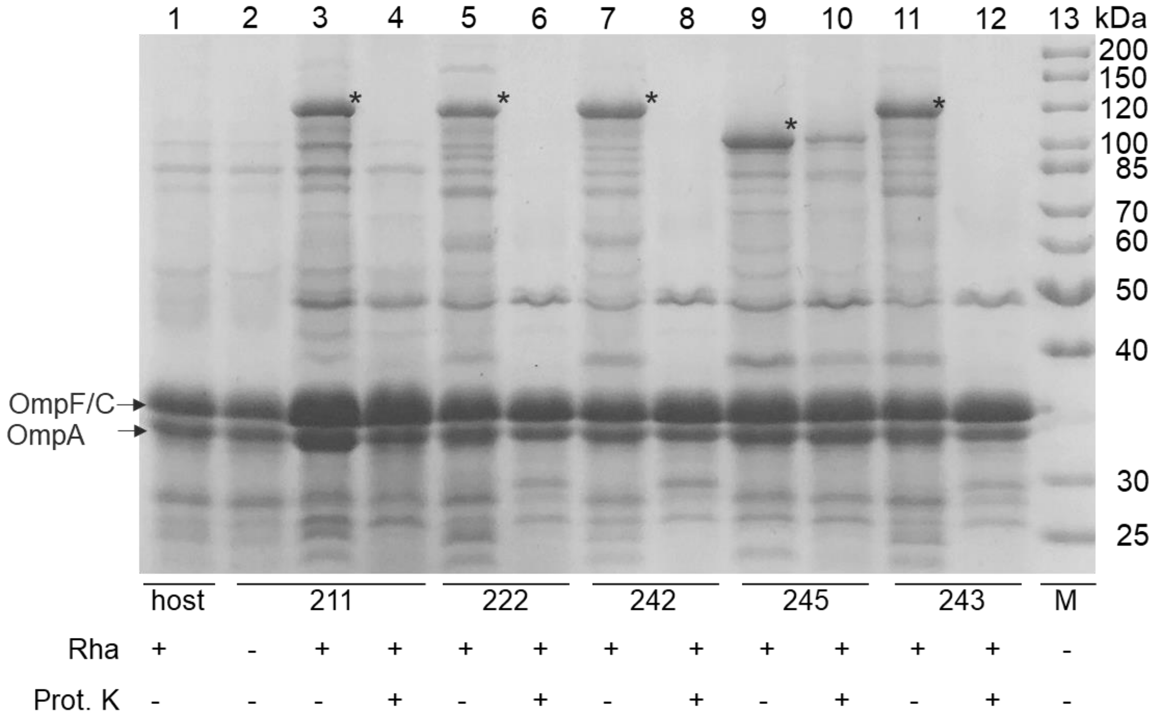

Supplementary Figure 1: Analysis of β-Gluc surface display level. *Membrane protein isolations (MPI) of E. coli host cells (lane 1) and E. coli pATB-β-Gluc variants P_{rhaBAD}-ctxB-β-gluc-epitope-ehaA (211, 111.9 kDa, reference), P_{rhaBAD}-oprF-β-gluc-flex-ehaA (222, 110.8 kDa), P_{rhaBAD}-oprF-spacer-β-gluc-flex-ehaA (242, 111.7 kDa), P_{rhaBAD}-oprF-spacer-β-gluc-ΔepitopeΔβ1-ehaA (245, 97.2 kDa), P_{rhaBAD}-oprf-spacer-β-gluc-rigid-ehaA (243, 112.6 kDa) (lane 2 – 12) were analyzed via SDS PAGE followed by Coomassie Brilliant Blue staining. The respective pATB code and the calculated molecular weight is given in brackets. The samples encompassed cells cultivated in 200 mL LB medium in 1 L shake flask scale with (lanes 1 & 3 -12) or without (lane 2) addition of 2 mM rhamnose (final concentration, Rha) as inductor at cell suspension OD_{578nm} 0.5. Prior to the MPI, one fraction of cells per variant was subjected to proteinase K (Prot. K) digest (lane 4, 6, 8, 10, 12), to approve surface accessibility of β-Gluc. Since Prot. K cannot penetrate the outer membrane of intact cells, protein digest is limited to proteins presented on the surface. Bands corresponding to the endogenous outer membrane protein OmpA (35 kDa) were unaffected and none additional bands corresponding to digested OmpA occurred in these samples. OmpA encompasses a C-terminal, periplasmatic domain that can be proteolytically digested, if Prot. K has access to the periplasm. Unaffected OmpA indicated intact cell membranes. Asterisks: supposed AT-FP bands. M: protein marker.*

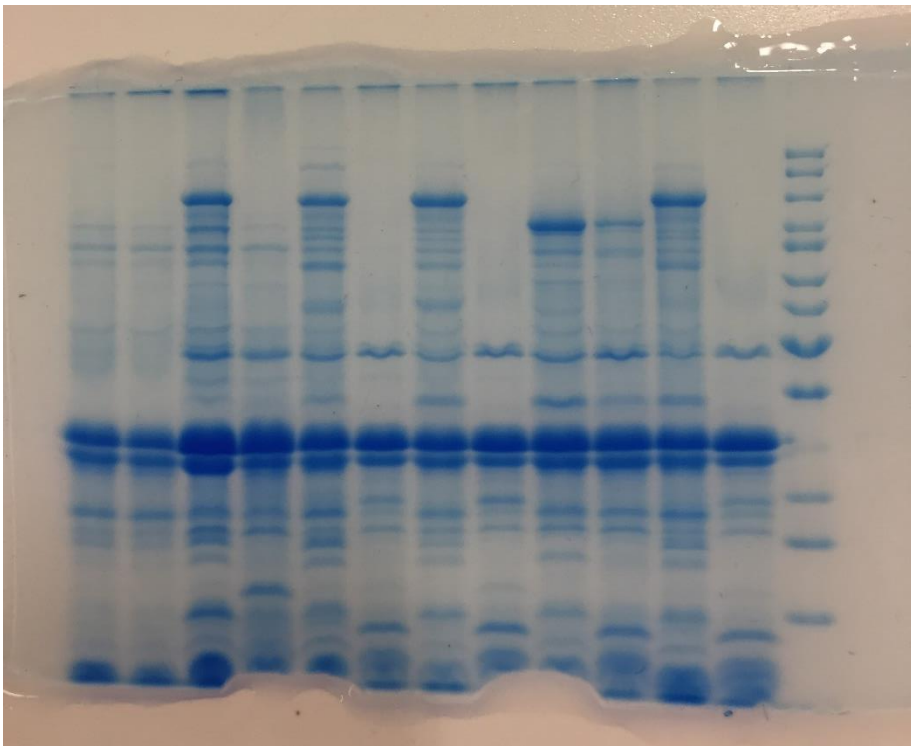

Supplementary Figure 2: Uncropped and unedited gel image of the gel shown in Supplementary Figure1.

Supplementary Table 1: Densitometric analysis of the SDS PAGE shown in Supplementary Figure 1. *For the separation of outer membrane fractions of E. coli pATB-β-Gluc variants P_{rhaBAD}-oprF-β-gluc-flex-ehaA (222), P_{rhaBAD}-oprF-spacer-β-gluc-flex-ehaA (242), P_{rhaBAD}-oprf-spacer-β-gluc-rigid-ehaA (243), P_{rhaBAD}-oprF-spacer-β-gluc-ΔepitopeΔβ1-ehaA (245), the reference P_{rhaBAD}-ctxB-β-gluc-epitope-ehaA (211) and E. coli host cells. The respective pATB code is given in brackets.*

1. Using the software ImageJ, intensities were determined for the bands of supposed autotransporter fusion proteins (AT-FP) and OmpA of each lane of the SDS gel (C6-G6 & C7-G7).

2. The higher molecular weight (MW) of AT-FP compared to OmpA was taken into account (C8-G8).

3. OmpA served as internal standard with an assumed number of 10^5 molecules per cell[1] (B11).

4. Using the estimated number of OmpA molecules per cell (B11), the ratio of AT-FP & OmpA band intensities (C8-G8) and the ratio of AT-FP & OmpA MWs (C10-G10), the β-Gluc molecules per cell for each variant were calculated (C11-G11).

5. The relative differences of β-Gluc molecules per cell compared to the reference 211 were calculated for each variant (C12-G12).

	A	B	C	D	E	F	G
1	**Lane**		**3**	**5**	**7**	**9**	**11**
2	Variant		211	222	242	245	243
3	Reference for enzyme count	OmpA					
4	Molecular weight (MW) (in kDa)	35,18	111.95	110.84	111.66	97.2	112.62
5	**Determination of intensities from SDS gel**						
6	Band intensity of supposed AT-FP		8,919,447	6,870,012	8,404,790	10,144,518	9,066,962
7	Band intensity of OmpA		19,508,912	16,843,255	14,527,376	13,402,305	14,936,426
8	Ratio AT-FP & OmpA band intensities		0.457	0.408	0.579	0.757	0.607
9	**Determination of molecules per cell**						
10	Ratio AT-FP & OmpA MWs		3.182	3.151	3.174	2.763	3.201
11	β-Gluc molecules per cell	100000	14,367	12,946	18,228	27,396	18,963
12	Relative differences compared to reference 211		100%	90%	127%	191%	132%

Supplementary Table 2: Comparison of pATB β-Gluc best performing variants. *The data were obtained from the activity verification with pNPG (yellow) as shown in Figure 5a of the 'results' section and from staining with Dylight633-conjugated antibody (orange) as shown in Figure 5f of the 'results' section. Data from both experiments were used to calculate the activity with pNPG normalized to the degree of surface display (grey). P_{rhaBAD}-ctxB-β-gluc-epitope-ehaA (211, reference), P_{rhaBAD}-oprF-β-gluc-flex-ehaA (222), P_{rhaBAD}-oprF-spacer-β-gluc-flex-ehaA (242), P_{rhaBAD}-oprf-spacer-β-gluc-rigid-ehaA (243), P_{rhaBAD}-oprF-spacer-β-gluc-ΔepitopeΔβ1-ehaA (245). The respective pATB code is given in brackets.*

Variant	Activity with pNPG [mU/mLOD$_{578nm}$]	mFI induced	mFI not induced	relative mFI (RmFI)	relative mFI relative to reference 211	Activity with *p*NPG normalized to RmFI
211	193.6	2878	533	5.4	1.00	35.8
222	703	2525	536	4.7	0.87	149.6
242	708.3	2458	475	5.2	0.96	136.2
243	571.5	2439	549	4.4	0.82	129.9
245	948.2	2564	570	4.5	0.83	210.7

Supplementary Table 3: Comparison of pATB HCN2-CNBD best performing variants. *The data were either obtained from 8-Fluo-cAMP binding assay (green) as shown in 'results' section Figure 9g or from staining with Dylight633-conjugated antibody (orange) as shown in 'results' section Figure 9h. Data from both experiments were used to normalize the 8-Fluo-cAMP binding capacity by degree of display (grey). P_{araBAD}-oprF-hcn2-cnbd-Δepitope-ehaA (124), P_{araBAD}-oprF-hcn2-cnbd-ΔepitopeΔβ1-ehaA* (125) and *P_{araBAD}-oprF-spacer-hcn2-cnbd-epitope-ehaA (141). The respective pATB code is given in brackets.*

Passenger	Variant	8-Fluo-cAMP binding capacity (mFI)	mFI induced	mFI not induced	Relative mFI (RmFI)	8-Fluo-cAMP binding capacity normalized to RmFI
HCN2-CnBD	124	1672	7018	193	40.8	40.9
	125	751	1812	174	10.5	75.1
	141	1363	9704	173	56.4	24.1

Supplementary Table 4: Quantification of the effect of different linkers on the activity of variants with identical SP based on step-by-step raw data.

	**Compared pATB variants[2]**	**Difference of mean activities[3]**	**Significantly different? (Level of significance)[4]**
Variants with P_{rhaBAD} (2yz) & CtxB SP (x1z) but different linker (xy1-6)	211 vs 212	-0.9334	Yes (*)
	211 vs 213	-0.3634	No
	211 vs 214	-0.2747	No
	211 vs 215	-0.895	No
	211 vs 216	0.4492	No
	212 vs 213	0.57	No
	212 vs 214	0.6587	No
	212 vs 215	0.03834	No
	212 vs 216	1.383	Yes (**)
	213 vs 214	0.08872	No
	213 vs 215	-0.5316	No
	213 vs 216	0.8127	No
	214 vs 215	-0.6203	No
	214 vs 216	0.7239	No
	215 vs 216	1.344	Yes (**)
Variants with P_{rhaBAD} (2yz) & OprF (x2z) but different linker (xy1-6)	221 vs 222	-7.869	Yes (***)
	221 vs 223	0.09874	No
	221 vs 224	-1.316	No
	221 vs 225	-2.426	Yes (**)
	221 vs 226	-0.01577	No
	222 vs 223	7.968	Yes (***)
	222 vs 224	6.554	Yes (***)
	222 vs 225	5.444	Yes (***)
	222 vs 226	7.853	Yes (***)
	223 vs 224	-1.414	No
	223 vs 225	-2.524	Yes (**)

Continuation of variants with P_{rhaBAD} (2yz) & OprF (x2z) but different linker (xy1-6)	**Compared pATB variants[2]**	**Difference of mean activities[3]**	**Significantly different? (Level of significance)[4]**
	223 vs 226	-0.1145	No
	224 vs 225	-1.11	No
	224 vs 226	1.3	No
	225 vs 226	2.41	Yes (**)
Variants with P_{rhaBAD} (2yz) & CtxB-spacer (x3z) but different linker (xy1-6)	231 vs 232	-1.433	Yes (**)
	231 vs 233	-0.5939	No
	231 vs 234	-0.4352	No
	231 vs 235	-0.6418	No
	231 vs 236	0.2899	No
	232 vs 233	0.839	No
	232 vs 234	0.9976	No
	232 vs 235	0.791	No
	232 vs 236	1.723	Yes (***)
	233 vs 234	0.1587	No
	233 vs 235	-0.04792	No
	233 vs 236	0.8838	No
	234 vs 235	-0.2066	No
	234 vs 236	0.7251	No
	235 vs 236	0.9317	No
Variants with P_{rhaBAD} (2yz) & OprF-spacer (x4z) but different linker (xy1-6)	241 vs 242	-4.445	Yes (*)
	241 vs 243	-2.202	No
	241 vs 244	-1.453	No
	241 vs 245	-7.918	Yes (***)
	241 vs 246	-0.4	No
	242 vs 243	2.243	No
	242 vs 244	2.991	No
	242 vs 245	-3.473	No
	242 vs 246	4.045	Yes (*)

Continuation of variants with P_{rhaBAD} (2yz) & OprF-spacer (x4z) but different linker (xy1-6)	**Compared pATB variants[2]**	**Difference of mean activities[3]**	**Significantly different? (Level of significance)[4]**
	243 vs 244	0.7485	No
	243 vs 245	-5.716	Yes (**)
	243 vs 246	1.802	No
	244 vs 245	-6.464	Yes (***)
	244 vs 246	1.053	No
	245 vs 246	7.518	Yes (***)

[1]z = linker = 1 (epitope) or 2 (flex) or 3 (rigid) or 4 (∆epitope) or 5 (∆epitope∆β1) or 6 (∆epitope∆β1∆CR)
[2]n = 4 biologically independent samples, one-way ANOVA, post-test Bonferroni multiple comparison
[3]Difference of mean activities = mean activity of first variant - mean activity of second variant (e.g. Difference of mean activities = mean activity of 211 - mean activity of 212)
[4]No = not significant with $p > 0.05$, Yes = significant with *$p \leq 0.05$, **$p \leq 0.01$, ***$p \leq 0.001$

Supplementary Table 5: Quantification of the effect of different SPs on variant activity based on step-by-step raw data.

	**Compared pATB variants[2]**	**Difference of mean activities[3]**	**Significantly different? (Level of significance)[4]**
Variants with P_{rhaBAD} (2yz) & same linker (xy1-6) but CtxB (x1z, x3z) or OprF SP (x2z, x4z)	211 vs 221	-1,439	No
	212 vs 222	-8,375	Yes (***)
	213 vs 223	-0,9768	No
	214 vs 224	-2,480	Yes (**)
	215 vs 225	-2,970	Yes (***)
	216 vs 226	-1,904	Yes (*)
	231 vs 241	-2,723	Yes (***)
	232 vs 242	-5,735	Yes (***)
	233 vs 243	-4,331	Yes (***)
	234 vs 244	-3,741	Yes (***)
	235 vs 245	-9,999	Yes (***)
	236 vs 246	-3,413	Yes (***)

[1]z = linker = 1 (epitope) or 2 (flex) or 3 (rigid) or 4 (∆epitope) or 5 (∆epitope∆β1) or 6 (∆epitope∆β1∆CR)
[2]n = 4, biologically independent samples, one-way ANOVA, post-test Bonferroni multiple comparison
[3]Difference of mean activities = mean activity of first variant - mean activity of second variant (e.g. Difference of mean activities = mean activity of 211 - mean activity of 221)
[4]No = not significant with $p > 0.05$, Yes = significant with *$p \leq 0.05$, **$p \leq 0.01$, ***$p \leq 0.001$

Supplementary Table 6: Quantification of the effect of the spacer on variant activity based on step-by-step raw data.

	**Compared pATB variants[2]**	**Difference of mean activities[3]**	**Significantly different? (Level of significance)[4]**
Variants with P_{rhaBAD} (2yz), same linker (xy1-6) & SP without (x1z, x2z) or with spacer (x3z, x4z)	211 vs 231	0.01883	No
	212 vs 232	-0.4807	No
	213 vs 233	-0.2117	No
	214 vs 234	-0.1417	No
	215 vs 235	0.272	No
	216 vs 236	-0.1405	No
	221 vs 241	-1.265	No
	222 vs 242	2.159	Yes (**)
	223 vs 243	-3.566	Yes (***)
	224 vs 244	-1.403	No
	225 vs 245	-6.757	Yes (***)
	226 vs 246	-1.649	No

[1]z = linker = 1 (epitope) or 2 (flex) or 3 (rigid) or 4 (Δepitope) or 5 (ΔepitopeΔβ1) or 6 (ΔepitopeΔβ1ΔCR)
[2]n = 4, biologically independent samples, one-way ANOVA, post-test Bonferroni multiple comparison
[3]Difference of mean activities = mean activity of first variant - mean activity of second variant (e.g. Difference of mean activities = mean activity of 211 - mean activity of 231)
[4]No = not significant with $p > 0.05$, Yes = significant with *$p \leq 0.05$, **$p \leq 0.01$, ***$p \leq 0.001$

```
CTCGAGGTTCCGCCGCTGTCTGTTCAGGGTAACAAAGTTGTTTCTGGTGGTCAGCAGGTTTCTCTGGGTGGTAACTCTCTGTTCTGGTCTAACAACGGT
TGGGGTGGTGAACGTTTCTACAACTCTGGTGCTGTTGGTGCTATCAAAAACGACTGGAAATCTTCTATCGTTCGTGCTGCTATGGGTGTTGACGAAGGT
GGTGGTTACCTGCAGGACCGTGAAGGTAACCGTAACAAAGTTATCTCTGTTGTTGACGCTGCTATCGCTAACGACATGTACGTTATCATCGACTGGCAC
TCTCACCACGCTCACCAGTACAAAAACGAAGCTATCGAATTCTTCCAGGACATGGCTCGTCGTTACGGTGACAAAAACAACGTTATCTACGAAGTTTAC
AACGAACCGCTGGACGTTTCTTGGTCTGGTGTTATCAAACCGTACGCTGAAGCTGTTATCGACGCTATCCGTCAGGTTGACCCGGACAACCTGATCATC
GTTGGTACTCGTCAGTGGTCTCAGGAAGTTGAAGAAGCTTCTTGGGACCCGATCCGTAAAAACAACATCGCTTACACCCTGCACTTCTACGCTGGTACT
CACAAACAGTGGCTGCGTGACAAAGCTCAGAACGCTATGAACAACGGTATCGCTCTGTTCGTTACCGAATGGGGTACTGTTGACGCTTCTGGTGACGGT
GCTGTTAACGAATCTGAAACCTGGGCTTGGGTTGACTTCATGCGTAACCACGGTATCTCTCACGCTAACTGGGCTCTGAACGACAAAGCTGAAGGTGCT
TCTACCTTCTGGCCGGGTGCTTCTGGTACTGGTGGTTGGAACGACGGTAACCTGACCCCGTCTGGTAAACTGGTTAAATCTATCATCCAGTCTTCTGAC
CCGATCCCGGGTGGTGACGACCCGGGTCCGGGTCCGGACTGCGGTTCTGTTTCTGCTCCGGGTAAAGTTCAGGCTGAAAACTACTGCGAAATGGAAGGT
GTTGAAAAAGAAAACACCTCTGACGCTGGTGGTGGTCAGAACCTGGGTTACATCGACTCTGGTGACTGGATGACCTACAAAATCAACGTTCCGTCTAAC
GGTGTTTACACCCTGTCTTACCGTGTTGCTTCTCTGAACGGTGGTGGTATCATCCAGGCTGAAAAAGCTGGTGGTTCTCCGGTTTACGGTTCTGTTGAA
ATCCCGTCTACCGGTGGTTGGCAGAACTGGAAAACCATCTCTCACGACATCCAGCTGTCTGCTGGTGAACAGCGTATCGGTCTGGCTGCTGTTTCTGGT
GGTTTCAACCTGAACTGGTTCGACGTTACCCAGAAAGGTGGTCCGGCTCCGAACGCTATCACCGTTCAGGCTGAAGAATACCTGGTTATGTCTGGTGTT
GAACTGGAAAACACCTCTGACGCTGGTGGTGGTAAAAACGTTGGTTACATCGACGCTAACGACTGGATGTCTTACCCGGAAGTTGACATCCCGGAATCT
GGTGTTTACACCGTTGAATACCGTGTTGCTTCTCTGTACGGTGGTGGTGTTATGCAGTTCGAAAAAGCTGGTGGTGACATCGTTTACGGTTCTGTTGAC
GTTCCGAACACCGGTGGTTGGCAGACCTGGAAAACCATCAAACACCAGGTTACCCTGGAAGCTGGTAAACAGCGTTTCGGTATCTACGCTCCGGCTGGT
GGTTGGAACCTGAACTGGTTCAAAATCACCAAAGGTCAGAAAGGTACC
```

Supplementary Figure 3: *Hahella chejuensis cel5 sequence (GenBank: ABC30636.1, Uniprot ID: Q2SFD8) codon optimized for E. coli.* *XhoI, KpnI restriction sites as added are underlined. This was the default passenger DNA sequence of all pATB.*

Supplementary Table 7: Sequences of oligonucleotides. pATBs denoted according to manuscript 'results' section Table 1.

Notation	Purpose	Nucleotide sequence (5` → 3`)
774 (fw)	Construction of pATB_121-*cel5*	CTCATCTCAGAAGAGGATCTGCTGACCAACAATGGCACG
770 (rv)	Construction of pATB_121-*cel5*	CTCTTCTGAGATGAGTTTTTGTTCTTTAAAGTATTCCGGGATGCG
3289 (fw)	Construction of pATB_131-*cel5,* *pATB*_141-*cel5,* pATB_231-*cel5,* pATB_241-*cel5*, pATB_331-*cel5*, pATB_341-*cel5*	CACCACCATCACCATCATATCGAAGGTCGT
3290 (fw)	Construction of pATB_131-*cel5* & PATB_141-*cel5*	GGCGGCGGTGACGACAACGCCGCGCCCGCCCACCACCATCACCAT
568 (fw)	Construction of pATB_211-*cel5*	ATGATCAAACTGAAATTCGGCGTCTTCTTC
984 (rv)	Construction of pATB_211-*cel5*	CGAGGCGGCTACAGCCGATAGTCTG
931 (fw)	Construction of pATB_211-*cel5*, pATB_221-*cel5* pATB_251-*cel5*, pATB_252-*cel5*, pATB_253-*cel5,*	GCTGTAGCCGCCTCGACATGTTCTTTCCTGCGTTA
534 (rv)	Construction of pATB_211-*cel5*	TTTCAGTTTGATCATATGTATATCTCCTTCTTAAGAATTGTTCAT
3291 (fw)	Construction of pATB_212-*cel5*	GGTGGAGGAGGTAGCGGCGGTTCAGGAGGTGGTGGATCCGGCGGTGGCGGTTCTGGTGGAGGTGGATCTCTGA CCAACAATGGCACGC
3292 (rv)	Construction of pATB_212-*cel5*	GCTACCTCCTCCACCGGTACCTTTCTGACC
2858 (fw)	Construction of pATB_213-*cel5*	GAGGCGGCAGCAAAGCTGACCAACAATGGCACGC

Notation	Purpose	Nucleotide sequence (5` → 3`)
2857 (rv)	Construction of pATB_213-*cel5*	CTTTGCTGCCGCCTCTTTAGCGGCGGCTTCCTTCGCTGCAGCCTCTTTCGCGGCCGCTTCTTTGGCTGCCGCTTCCG CGGTACCTTTCTGACCTTTGG
878 (fw)	Construction of pATB_214-*cel5*	CTGACCAACAATGGCACGC
879 (rv)	Construction of pATB_214-*cel5*	GCCATTGTTGGTCAGGGTACCTTTCTGACCTTTGGTGATTTTGAACCAG
883 (fw)	Construction of pATB_215-*cel5*	GGCCCGGAGCCTGATCCG
880 (rv)	Construction of pATB_215-*cel5*	ATCAGGCTCCGGGCCGGTACCTTTCTGACCTTTGGTGATTTTGAACCAG
814 (fw)	Construction of pATB_216-*cel5*	CCGACTCCGGGTCCGGACT
882 (rv)	Construction of pATB_216-*cel5*	CGGACCCGGAGTCGGGGTACCTTTCTGACCTTTGGTGATTTTGAACCAG
1584 (fw)	Construction of pATB_221-*cel5*	GAAGGAGATATACATATGAAACTGAAAAACACCTTGG
984 (rv)	Construction of pATB_221-*cel5*	CGAGGCGGCTACAGCCGATAGTCTG
1568 (rv)	Construction of pATB_221-*cel5*	ATGTATATCTCCTTCTTAAGAATTGTTCATTACG
3289 (fw)	Construction of pATB_331-*cel5*, pATB_341-*cel5*	CACCACCATCACCATCATATCGAAGGTCGT
3290 (rv)	Construction of pATB_331-*cel5*, pATB_341-*cel5*	GGCGGCGGTGACGACAACGCCGCGCCCGCCCACCACCATCACCAT

Notation	Purpose	Nucleotide sequence (5` → 3`)
841 (fw)	Verification of pATBs with P_{araBAD} *via* colony PCR, Verification of SP and β-barrel for pATB with P_{araBAD} *via* sequence analysis	GCGTCACACTTTGCTATGCC
565 (rv)	Verification of pATBs encoding epitope linker *via* colony PCR	TTTAAAGTATTCCGGGATGCGGCCCTC
2071 (rv)	Construction pATB_152-cel5, Verification of pATBs encoding flex linker *via* colony PCR	AGATCCACCTCCACCAGAAC
1075 (rv)	Verification of pATBs encoding Δepitope linker *via* colony PCR	GCCCTTAACAACCAGAACGTTACCC
349 (rv)	Verification of pATBs encoding ΔepitopeΔβ1 & ΔepitopeΔβ1ΔCR *via* colony PCR	CGCCTTTCACCGTATTATCG
2912 (rv)	Verification of pATBs encoding rigid linker *via* colony PCR	CTTTGCTGCCGCCTCTTTAG

Notation	Purpose	Nucleotide sequence (5` → 3`)
1863 (fw)	Verification of pATBs with P_{rhaBAD} *via* colony PCR, Verification of SP and β-barrel for pATB with P_{rhaBAD} *via* sequence analysis	GCATGCATCGATCACCACAA
1091 (rv)	Verification of pATBs with P_{Rox306} *via* colony PCR, Verification of SP and β-barrel for pATB with P_{Rox306} *via* sequence analysis	CGCACCCTGCCAGCCAGAC
3676 (fw)	Construction of pATB_152-*CsbglA*	GGTGGAGGTGGATCTCTCGAGAGCTTTCCGAAAG
3572 (rev)	Construction of pATB_152-*CsbglA*	GCTACCTCCTCCACCACGACCTTCGATATGATGGTGATG
1230 (fw)	Construction of pATB_152-*CsbglA*	GGTGGAGGAGGTAGCGGC
2071 (rev)	Construction of pATB_152-*CsbglA*	AGATCCACCTCCACCAGAAC
1815 (fw)	Construction of pATB_153-*CsbglA*	CTCGAGAGCTTTCCGAAAG

Notation	Purpose	Nucleotide sequence (5` → 3`)
915 (rev)	Construction of pATB_351-*CsbglA*, pATB_352-*CsbglA*, pATB_353-*CsbglA*	GACCGACCTCTCAGGTTTTTTATTGGAAG
2532 (rev)	Construction of pATB_153-*CsbglA*	ACGACCTTCGATATGATGGTGATG
2537 (fw)	Construction of pATB_153-*CsbglA*	CATATCGAAGGTCGTGCGGAAGCGGCAGCC
3573 (rev)	Construction of pATB_153-*CsbglA*	CGGAAAGCTCTCGAGCTTTGCTGCCGCCTCTTTAG
PR_ATB_01 (fw)	Construction of *Bacillus coagulans* CotA with XhoI restriction site	CTCGAGAGCCCGAATCTGGAAAAATTTG
PR_ATB_01 (rev)	Construction of *Bacillus coagulans* CotA with KpnI restriction site	GGTACCATCCAGCAGAGGACCATC
PR_ATB_01 (fw)	Construction of *Bacillus coagulans* CotA with XhoI/KpnI restriction site	CTCGAGAGCCCGAATCTGGAAAAATTTG

Notation	Purpose	Nucleotide sequence (5` → 3`)
PrMJ18-HCN2 (rev)	Construction of HCN2 C-Linker-CNBD with XhoI/KpnI restriction site	GGTACCATGCAGCAGAATGCTGTT
PR_ATB_01 (rev)	Construction of *Bacillus coagulans* CotA with XhoI/KpnI restriction site	GGTACCATCCAGCAGAGGACCATC
PrMJ01 (fw)	Construction of HCN2 C-Linker-CNBD with XhoI/KpnI restriction site	CTCGAGGATAGCAGCCGTCGTCAGTATC
Pr2018 (fw)	Verification of linker for pATB with YeeJ β-barrel *via* sequence analysis	GAAACCGATAACACCTGGCC
Pr2173 (rev)	Verification of linker for pATB with EhaA β-barrel *via* sequence analysis	CTCCGGACGCAGGTCGTTATC

Supplementary References

1. Koebnik, R., Locher, K.P. & Van Gelder, P. Structure and function of bacterial outer membrane proteins: Barrels in a nutshell. *Mol. Microbiol.* **37**, 239-253 (2000).
