# Supplementary material for: A plasmid toolbox for the easy autodisplay of recombinant proteins and its optimization
Source: Commun Biol. 2026 May 21;9:694. doi: 10.1038/s42003-026-10324-7 (PMC13195121; doi:10.1038/s42003-026-10324-7)
Supplement: Supplementary file 2 — Reporting Summary [file 42003_2026_10324_MOESM2_ESM.pdf]

nature portfolio

Corresponding author(s): Prof. Joachim Jose

Last updated by author(s): Apr 17, 2026

Reporting Summary

Nature Portfolio wishes to improve the reproducibility of the work that we publish. This form provides structure for consistency and transparency in reporting. For further information on Nature Portfolio policies, see our Editorial Policies and the Editorial Policy Checklist.

Statistics

For all statistical analyses, confirm that the following items are present in the figure legend, table legend, main text, or Methods section.

n/a	Confirmed	
☐	☒	The exact sample size (*n*) for each experimental group/condition, given as a discrete number and unit of measurement
☐	☒	A statement on whether measurements were taken from distinct samples or whether the same sample was measured repeatedly
☐	☒	The statistical test(s) used AND whether they are one- or two-sided<br>*Only common tests should be described solely by name; describe more complex techniques in the Methods section.*
☒	☐	A description of all covariates tested
☐	☒	A description of any assumptions or corrections, such as tests of normality and adjustment for multiple comparisons
☐	☒	A full description of the statistical parameters including central tendency (e.g. means) or other basic estimates (e.g. regression coefficient) AND variation (e.g. standard deviation) or associated estimates of uncertainty (e.g. confidence intervals)
☒	☐	For null hypothesis testing, the test statistic (e.g. *F*, *t*, *r*) with confidence intervals, effect sizes, degrees of freedom and *P* value noted<br>*Give P values as exact values whenever suitable.*
☒	☐	For Bayesian analysis, information on the choice of priors and Markov chain Monte Carlo settings
☒	☐	For hierarchical and complex designs, identification of the appropriate level for tests and full reporting of outcomes
☒	☐	Estimates of effect sizes (e.g. Cohen's *d*, Pearson's *r*), indicating how they were calculated

Our web collection on statistics for biologists contains articles on many of the points above.

Software and code

Policy information about availability of computer code

Data collection	Endnote version 20.0.1, Microsoft Excel version 2024,
Data analysis	Raptor X, version not specified, PEP-FOLD version 3, ImageJ version 1.50i, NEBiocalculator version 1.15.0, GLUE-IT CASTER version 2.0, GraphPad Prism version 6, Adobe Illustrator Artwork version 16.0, FlowJo version 10.8.1

For manuscripts utilizing custom algorithms or software that are central to the research but not yet described in published literature, software must be made available to editors and reviewers. We strongly encourage code deposition in a community repository (e.g. GitHub). See the Nature Portfolio guidelines for submitting code & software for further information.

Data

Policy information about availability of data

All manuscripts must include a data availability statement. This statement should provide the following information, where applicable:

- Accession codes, unique identifiers, or web links for publicly available datasets
- A description of any restrictions on data availability
- For clinical datasets or third party data, please ensure that the statement adheres to our policy

Data availability
Numerical source data for graphs and charts can be found on Figshare following the DOI 10.6084/m9.figshare.2696965975. All other data are available from the

corresponding author J.J. (joachim.jose@uni-muenster.de) on reasonable request. Data in this manuscript and its supplementary information are presented in aggregated form wherever possible. Single plasmids of the ATB, as well as pooled plasmid libraries can be obtained from Addgene (Watertown, USA).

Research involving human participants, their data, or biological material

Policy information about studies with human participants or human data. See also policy information about sex, gender (identity/presentation), and sexual orientation and race, ethnicity and racism.

Reporting on sex and gender	none
Reporting on race, ethnicity, or other socially relevant groupings	none
Population characteristics	none
Recruitment	none
Ethics oversight	none

Note that full information on the approval of the study protocol must also be provided in the manuscript.

Field-specific reporting

Please select the one below that is the best fit for your research. If you are not sure, read the appropriate sections before making your selection.

☒ Life sciences ☐ Behavioural & social sciences ☐ Ecological, evolutionary & environmental sciences

For a reference copy of the document with all sections, see nature.com/documents/nr-reporting-summary-flat.pdf

Life sciences study design

All studies must disclose on these points even when the disclosure is negative.

Sample size	Sample size was defined based on similar studies in this field.
Data exclusions	Figures of agarose gels that show each pATB backbone in the step-by-step cloning procedure.<br>Figures of SDS-PAGES of outer membrane proteins that were used to make an initial guess of the amount of protein loaded onto the SDS-PAGE.
Replication	The activity of all β-Gluc variants in the pATB step-by-step screening was determined as n = 4 biologically independent samples. The activity of β-Gluc variants with inversed composition, the verification of the highest performing variants of β-Gluc, CotA and HCN2-CNBD and the assessments of cell lysis was determined as n = 3 biologically independent samples. Each biological independent sample started with a bacterial colony picked from an agar plate. In the pATB-β-gluc all-in-one and strain-mix library screening, the activity of 105 variants were tested. In the pATB-cotA strain-mix library screening 90 variants were tested and in the pATB-hcn2-cnbd strain-mix library screening 110 (first round) and 24 (second round) were tested. Bacterial colonies were the result of a transformation with respective plasmids. A K-S test was performed with the cumulative frequency distributions of the pATB-β-gluc all-in-one and pATB-β-gluc strain-mix screening data sets. FACS Diva 8.0 was used for collecting the flow cytometry data with FACS Aria III device (Becton Dickinson, Heidelberg). To measure the Dylight633 fluorescence, an excitation wavelength of 633 nm and emission wavelength filters of 660/20 nm (band-pass) were used. GFP fluorescence was excited at 488 nm and measured with an emission wavelength filter of 530/30 nm (band-pass) and 502 nm (long-pass). No gating strategy was applied. FlowJo version 10.8.1 was used for analyzing the flow cytometry data. Surface accessibility of β-Gluc and HCN2-CNBD was determined by flow cytometry analysis measuring 50,000 cells per variant. Sequence analysis of high performing variants was done once per sequence of interest for each variant.
Randomization	Cells for the step-by-step, all-in-one and strain-mix library screening were randomly picked. For the verification of the highest performing variants and the analysis of their level of surface display randomization was not relevant because the strains were cultivated starting from uniform biological, i.e. bacterial colony or cryologically preserved stock of bacterial cells.
Blinding	Blinding of the investigator was not necessary for the all-in-one and strain-mix screening because the identity of the randomly chosen colonies were unknown during the experiment either way. The investigator was blinded for the step-by-step screening, the verification of the highest performing variants, for the analysis of level of surface display and for the assessment of cell integrity.

Reporting for specific materials, systems and methods

We require information from authors about some types of materials, experimental systems and methods used in many studies. Here, indicate whether each material, system or method listed is relevant to your study. If you are not sure if a list item applies to your research, read the appropriate section before selecting a response.

Materials & experimental systems

n/a	Involved in the study	
☐	☒	Antibodies
☒	☐	Eukaryotic cell lines
☒	☐	Palaeontology and archaeology
☒	☐	Animals and other organisms
☒	☐	Clinical data
☒	☐	Dual use research of concern
☒	☐	Plants

Methods

n/a	Involved in the study	
☒	☐	ChIP-seq
☐	☒	Flow cytometry
☒	☐	MRI-based neuroimaging

Antibodies

Antibodies used

The following commercially-available antibodies were used in this study. Respective catalog, clone and lot numbers are provided wherever, this information was publicly accessible. Antibody dilutions are given in 'Methods' section of the manuscript.

1. Monoclonal mouse Anti-His6 antibody, producer: Genscript, distributor: Antikoerper-online, catalog number: ABIN387699, clone number: 6G2A9

2. DyLight 633 conjugated goat anti-mouse IgG (H + L) antibody, producer/distributor: Thermo Fisher, catalog number: 35512

Validation

The validation information for the antibodies listed above is available via the following links to the manufacturer's website.

1. https://www.antikoerper-online.de/antibody/387699/anti-His+Tag+antibody/

2. https://www.thermofisher.com/antibody/product/Goat-anti-Mouse-IgG-H-L-Secondary-Antibody-Polyclonal/35512

Plants

Seed stocks

none

Novel plant genotypes

none

Authentication

none

Flow Cytometry

Plots

Confirm that:

☒ The axis labels state the marker and fluorochrome used (e.g. CD4-FITC).

☒ The axis scales are clearly visible. Include numbers along axes only for bottom left plot of group (a 'group' is an analysis of identical markers).

☐ All plots are contour plots with outliers or pseudocolor plots.

☒ A numerical value for number of cells or percentage (with statistics) is provided.

Methodology

Sample preparation

The sample preparation is described in the 'Methods' section of the manuscript.

Instrument

FACS Aria III (Becton Dickinson, Heidelberg)

Software

FACS Diva 8.0 for collecting the flow cytometry data and FlowJo version 10.8.1 for analyzing the flow cytometry data.

Cell population abundance

The flow cytometry was used to quantify the level of protein displayed at the bacterial surface.

Gating strategy	The flow cytometry was used to quantify the level of protein displayed at the bacterial surface. Therefore, no gating strategy was applied.

☐ Tick this box to confirm that a figure exemplifying the gating strategy is provided in the Supplementary Information.
